# Enhancement of anaerobic glycolysis – a role of PGC-1α4 in resistance exercise

**DOI:** 10.1038/s41467-022-30056-6

**Published:** 2022-04-28

**Authors:** Jin-Ho Koh, Mark W. Pataky, Surendra Dasari, Katherine A. Klaus, Ivan Vuckovic, Gregory N. Ruegsegger, Arathi Prabha Kumar, Matthew M. Robinson, K. Sreekumaran Nair

**Affiliations:** 1 Division of Endocrinology and Metabolism, Mayo Clinic, Rochester, MN United States; 2 Department of Physiology, College of Medicine, Yeungnam University, Daegu, Korea; 3 Department of Health Sciences Research, Mayo Clinic, Rochester, MN United States; 4 Mayo Clinic Regional Comprehensive Metabolomics Core, Mayo Clinic, Rochester, MN United States; 5 School of Biological and Population Health Sciences, College of Public Health and Human Sciences, Oregon State University, Corvallis, OR 97331 United States

**Keywords:** Skeletal muscle, Metabolism

## Abstract

Resistance exercise training (RET) is an effective countermeasure to sarcopenia, related frailty and metabolic disorders. Here, we show that an RET-induced increase in PGC-1α4 (an isoform of the transcriptional co-activator PGC-1α) expression not only promotes muscle hypertrophy but also enhances glycolysis, providing a rapid supply of ATP for muscle contractions. In human skeletal muscle, PGC-1α4 binds to the nuclear receptor PPARβ following RET, resulting in downstream effects on the expressions of key glycolytic genes. In myotubes, we show that PGC-1α4 overexpression increases anaerobic glycolysis in a PPARβ-dependent manner and promotes muscle glucose uptake and fat oxidation. In contrast, we found that an acute resistance exercise bout activates glycolysis in an AMPK-dependent manner. These results provide a mechanistic link between RET and improved glucose metabolism, offering an important therapeutic target to counteract aging and inactivity-induced metabolic diseases benefitting those who cannot exercise due to many reasons.

## Abstract (web summary)

Resistance exercise training (RET) is an effective countermeasure to sarcopenia, related frailty and metabolic disorders. Here, the authors show that an RET-induced increase in PGC-1α4 expression not only promotes muscle hypertrophy but also enhances glycolysis, providing a rapid supply of ATP for muscle contractions.

## Introduction

Muscle weakness and wasting (sarcopenia) are major emerging public health concerns that commonly occur as people age^1,2^ and are even more prevalent in older patients with type 2 diabetes (T2DM)^3,4^. Sarcopenia is associated with not only increased rates of frailty, but also cardiometabolic diseases including insulin-resistant states such as T2DM and dementia^5,6^. Clinical studies have revealed that resistance exercise training (RET) improves not only muscle mass and strength, but also glucose and lipid disposal in people with T2DM or those at risk for cardiometabolic diseases^7–12^. However, unlike aerobic exercise training (AET), which primarily uses energy supplied from mitochondrial oxidative metabolism^13,14^, less is known about energy metabolism and its regulation in contracting muscle during resistance exercise (RE). The prevailing data support the notion that the ATP-PCr system and anaerobic glycolysis are the critical energy sources for RE^15,16^, with anaerobic glycolysis contributing the majority of ATP after the first 5 sec of RE. Although RET has a minimal to moderate effect on mitochondrial biogenesis and function^17^, especially compared to AET^18^, various other physiological and morphological health benefits occur associated with RE including muscle hypertrophy, strength and improved glucose metabolism^7–12^, which act as countermeasures to T2D and related metabolic disorders. However, a substantial number of people with orthopedic pain and/or other disabilities are unable to perform RE and thus cannot benefit from RET. The underpinning mechanisms of these beneficial effects of RET will allow targeted therapeutic research to develop pharmaceutical products that potentially benefit those who cannot engage in RET.

After the peroxisome proliferator-activated receptor co-activator-γ-1α (PGC-1α) was revealed as a transcriptional co-activator that orchestrates mitochondrial biogenesis, numerous PGC-1α-mediated metabolic mechanisms in muscle were studied. Interestingly, despite RET-induced increases in PGC-1α^19^, mitochondrial biogenesis and adaptive increases in oxidative phosphorylation by RET are substantially muted compared to AET^17,18,20–22^. Moreover, the metabolic and physiological phenotypes of muscle following each of these exercise-training modes are quite different^21,22^. Multiple isoforms of PGC-1α exist. PGC-1α1, driven by the proximal promotor, constitutes the majority of PGC-1α isoform mRNA expression in many tissues, including liver, kidney, and brown adipose tissue^19^, thus PGC-1α1 is by far the most studied isoform and is often identified simply as “PGC-1α” (instead of PGC-1α1). In muscle, the mRNA expression levels of other PGC-1α isoforms (including PGC-1α2, -1α3, and -1α4) more closely resemble PGC-1α1^19^. In myotubes, overexpression of PGC-1α1 or PGC-1α4 (a spliced variant driven by the alternative promotor) drive the majority of gene changes, whereas PGC-1α2 and PGC-1α3 affect far fewer genes and their function is presently unknown^19^. Previous studies have shown that endurance exercise increases PGC-1α1 gene expression and promotes an oxidative phenotype^23,24^, whereas RE increases gene expression of the truncated isoform, PGC-1α4, promoting muscle hypertrophy^19^. It appears that the PGC-1α isoform genes can be differently regulated by the type of muscular contraction. Since resistance exercise enhances glucose metabolism through mechanisms that are apparently independent of increases in aerobic metabolism and the PGC-1α1 isoform, we hypothesized that in addition to enhancing muscle hypertrophy, that PGC-1α4 also regulates alterations in anaerobic energy metabolism by RE.

Since PGC-1α isoforms are transcriptional coactivators, they need to interact with both a sensor recognizing cellular energy status and a nuclear receptor to govern bioenergetics. Peroxisome proliferator-activated receptor β (PPARβ) is a dominant isoform of the PPAR nuclear receptor family in skeletal muscle^25^, and 5’AMP-activated protein kinase (AMPK) is a critical energy sensor in skeletal muscle^26^. PGC-1α1 has previously been shown to cooperate with AMPK and PPARβ to regulate glucose uptake^27^, but the interaction of PGC-1α4 with AMPK and PPARβ is unknown. Since RET improves glucose metabolism, we hypothesized that the RET-induced spliced variant PGC-1α4 also interacts with AMPK and PPARβ to orchestrate enhanced glucose metabolism.

Here, we identified that PGC-1α4, induced by RE, likely facilitates glycolysis and glucose uptake in muscle via enhanced glycolytic enzymes. We also observed that PGC-1α4 cooperates with PPARβ and AMPK to regulate glucose metabolism. Identifying therapies or drugs that can target these molecular pathways will likely advance our ability to prevent/treat metabolic disease, especially in those who are unable to perform RE.

## Results

### Resistance exercise upregulates glycolysis in human skeletal muscle

**Fig. 1:**
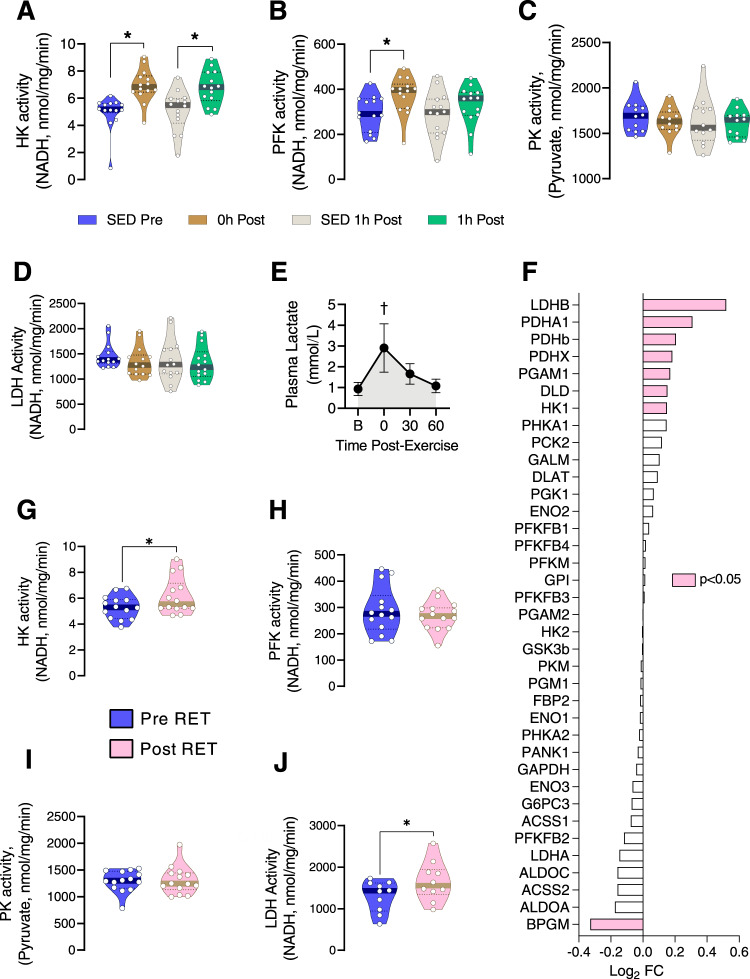
Resistance exercise upregulates glycolysis in human skeletal muscle. **A**–**D** The enzyme activity of hexokinase (HK), phosphofructokinase (PFK), pyruvate kinase (PK), and lactate dehydrogenase (LDH) were analyzed from muscle biopsies of healthy participants before (SED Pre), within 10 min after (0 h Post), and 1 h after (1 h Post) a one-legged resistance exercise (RE) bout (*n* = 14 for HK, PFK and LDH; *n* = 12 for PK). Time-matched control muscle samples were obtained from the non-exercised leg at 1 h post-RE (SED Post). *P < 0.05 vs. SED in each group. One-way ANOVA was used with multiple comparisons. **E** Plasma lactate was measured at baseline (B), immediately post-exercise (0), 30 min post-exercise (30), and 60 min post-exercise (60) to represent the “spill over” of lactate from muscle to blood (*n* = 17). † indicates a significant difference between the immediate post-exercise and baseline lactate samples (*P* < 0.05). **F** RNA-Seq data for glycolysis-related genes in muscle after resistance exercise training (RET) (*n* = 18). Data were analyzed from RNA-Seq results presented in our previous publication^18^. **G**–**J** Muscles were obtained before (pre-RET) and after (post-RET) 12 weeks of RET and subsequently analyzed for HK, PFK, PK, and LDH activity (*n* = 14 for HK, PFK, and PK; *n* = 10 for LDH). *P < 0.05 vs. pre-RET. Paired two-tailed *t*-test was used. Values are expressed as individual data points, Log_2_ FC, or mean ± SD. Significant labeled *P*-values in each panel from left to right are as follows: panel **A** = 0.001 and 0.007; panel **B** = 0.004; panel **E** = < 0.001; panel **G** = 0.009; panel **J** = 0.004.

There is a need of rapid ATP supply in contracting skeletal muscle during RE that cannot be provided by oxidative phosphorylation, which involves multiple metabolic steps including the citric acid cycle and the electron transport chain. It is known that a single session of resistance exercise increases the rate of energy utilization in skeletal muscle through enhanced glycogenolysis and phosphagen breakdown^28^. Here, based on one-legged resistance exercise, we provide further support that RE enhances glycolysis, enabling a greater capacity for rapid ATP production in skeletal muscle, which is not evident in the contralateral non-exercised leg. Participants (Supplemental Table 1) performed a single session of one-legged knee-extension RE. Muscle biopsies were obtained before and after the acute exercise bout as noted in the methods. The activity of hexokinase (HK), the critical first enzyme in the glycolytic pathway which converts glucose to glucose-6 phosphate^29^, was elevated immediately after RE in skeletal muscle and remained elevated up to 1 h post-exercise (Fig. 1A). The activity of phosphofructokinase (PFK), another rate-limiting enzyme in glycolysis, was also increased immediately after RE, but returned to baseline by 1 h post-exercise (Fig. 1B). However, the activity of pyruvate kinase (PK), the furthest downstream enzyme of the glycolytic pathway^30^, was unaltered by acute RE (Fig. 1C). Although we found no change in the activity of lactate dehydrogenase (LDH) after acute RE (Fig. 1D), we found that plasma lactate was rapidly elevated after acute RE (Fig. 1E), consistent with the idea that lactate is produced by glycolysis during muscle contraction^31^. Together, these data provide strong evidence that glycolysis is elevated in skeletal muscle after a single bout of RE by enhanced activity of the rate-limiting glycolytic enzymes HK and PFK. Our results are consistent with a previous study in rodents which showed that in transgenic mice, which display reduced glycolytic capacity, HK and PFK activity were reduced, but PK did not change^30^. Of note, HK activity at 1 h post-exercise was unaltered in the non-exercised leg, providing evidence that the post-RE increase in muscle glycolysis is not systemic, but is limited to exercised muscles.

As we observed increased markers of glycolysis after one bout of RE in skeletal muscle, we sought to determine whether adaptations to enhance glycolysis occur in skeletal muscle following 12 weeks of RET. In our previous study, participants completed 12 weeks of supervised progressive RET, which resulted in increased muscle mass, and insulin sensitivity^18^. We used muscle biopsies before RET (Pre-RET) and 72 h following the last bout of RET (Post-RET) collected from our previous study. Genes related to glucose metabolism including HK1, PGAM1, PDH, PDHA1 and LDHB were higher post-RET compared to pre-RET (Fig. 1F). We also found that acute RE rapidly increases mRNA expression of some glycolytic enzymes (HKII, PFK, and aldolase) (Supplemental Fig. 1), but it is highly unlikely that changes in protein expression can occur quickly enough after acute RE to result in any functional changes in glycolysis. The activity of HK (Fig. 1G), but not PFK (Fig. 1H) or PK (Fig. 1I), was significantly increased in skeletal muscle after RET in biopsy samples collected 72 h following last bout of RE. This observation is consistent with Tang et al., who showed that after a similar 12-week RET protocol HK activity, but not PFK activity, is increased in skeletal muscle^32^. Similarly, Hjeltnes et al. found increased HKII activity, but not PFK activity in skeletal muscle from participants with tetraplegia after 8 weeks of electrical stimulation, concomitant with increased HKII abundance^33^. Moreover, we found an increase in LDH activity following RET (Fig. 1J). Together, these results suggest that either acute RE or chronic RET can enhance glycolysis in human skeletal muscle. Acute RE likely enhances glycolytic enzyme activity via post-translational mechanisms because protein synthesis cannot occur quickly enough to accommodate such a rapid increase in glycolytic enzyme abundance. Likewise, it is possible that an increase in glycolytic enzyme abundance following RET may contribute to the increase in glycolytic activity. We further investigated the mechanisms by which RE activates the glycolytic pathway.

### Resistance exercise increases PGC-1α4 expression, which is correlated with glycolytic gene expression in human skeletal muscle

**Fig. 2:**
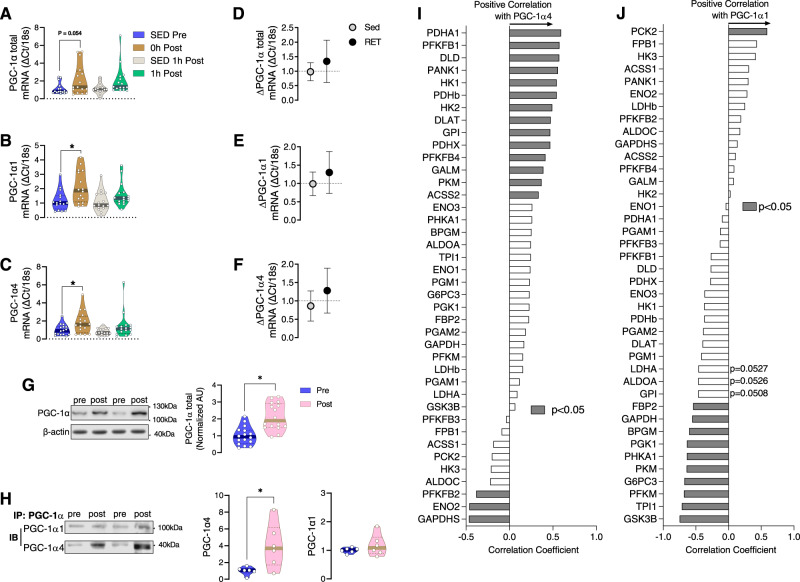
Resistance exercise increases PGC-1α4 expression which is correlated with glycolytic gene expression in human skeletal muscle. **A**–**C** Quantification of PGC-1α-total, PGC-1α1, and PGC-1α4 mRNA was done by qPCR using muscle biopsy samples of healthy participants before (SED Pre), within 10 min after (0 h Post), and 1 h after (1 h Post) a one-legged resistance exercise (RE) bout (*n* = 16). Time-matched control muscle samples were obtained from the non-exercised leg at 1 h post-RE (SED 1 h Post) (*n* = 16 per group). One-way ANOVA was used with multiple comparisons. **D**–**F** Participants performed resistance training (RET) for 12 weeks or remained sedentary (Sed) and muscle biopsies were obtained 72 h after the last bout of exercise. ΔPGC-1α total mRNA, ΔPGC-1α1 mRNA, and ΔPGC-1α4 mRNA were measured by qPCR before (Pre) and after (Post) 12 weeks of RET or Sed (*n* = 13 for PGC-1α total, PGC-1α1, and PGC-1α4 RET; *n* = 12 muscles for PGC-1α4 Sed). Unpaired two-tailed *t*-tests were used. **G** Protein abundance of total PGC-1α is displayed before and after RET (*n* = 16 per group). Paired two-tailed *t*-test was used. **H** Total PGC-1α was immunoprecipitated followed by immunoblotting for PGC-1α1 and PGC-1α4. Owing to the large amount of protein required to immunoprecipitate enough PGC-1α for immunoblot detection of PGC-1α1 and PGC-1α4, samples of multiple participants before and after training were pooled together, resulting in a smaller *n* for PGC-1α isoform quantification (*n* = 6). Unpaired two-tailed *t*-test was used. **I** Correlation between PGC-1α4 gene expression (measured by qPCR) and multiple glycolysis-related genes (measured by RNAseq) in skeletal muscle from sedentary participants (*n* = 35 from Robinson 2017 et al.; filled bars indicate significant correlation at *P* < 0.05). **J** Correlation between PGC-1α1 gene expression (measured by qPCR) and multiple glycolysis-related genes (measured by RNAseq) in skeletal muscle from sedentary participants (*n* = 35 from Robinson 2017 et al.; filled bars indicate significant correlation at *P* < 0.05). *P < 0.05. Values are expressed as individual data points, Log_2_ FC, or mean ± SD. Significant labeled *P*-values in each panel are as follows: panel **A** = 0.054; panel **B** = 0.04; panel **C** = 0.047; panel **G** = < 0.001; panel **H** = 0.023.

In a previous publication, we reported that RE-induced enhancement of the transcriptional co-activator PGC-1α4 results in muscle hypertrophy^19^. However, the influence of PGC-1α4 on other metabolic processes, such as the glycolytic pathway, is not yet known. We hypothesized that PGC-1α4 plays a key role in RE-induced increases in muscle glycolysis because RE enhances anaerobic metabolism, and PGC-1α4 has been implicated in other critical RE-mediated adaptations, such as hypertrophy and strength. First, we found that one bout of RE rapidly increased mRNA expression of total PGC-1α (Fig. 2A) and its isoforms PGC-1α1 (Fig. 2B) and PGC-1α4 (Fig. 2C) in human skeletal muscle, supporting the finding by Ydfors et al. that mRNA expression of both truncated (including PGC-1α4) and non-truncated (including PGC-1α1) forms of PGC-1α are induced by a single session of either aerobic or resistance exercise^34^. However, after 12 weeks of RET, the expression levels of all measured PGC-1α transcripts were unaltered when measured 72 h following the last exercise bout (Fig. 2D–F), suggesting a short half-life of PGC-1α transcript expression in skeletal muscle after acute RE. We determined the protein abundance of PGC-1α and its isoforms after acute and chronic RE training. Although PGC-1α isoform transcripts were elevated after acute RE, translational processes are not quick enough to elicit an increase in PGC-1α protein abundance in skeletal muscle by one hour after acute RE (Supplemental Fig. 2). However, after 12 weeks of RET the protein abundance of total PGC-1α was significantly elevated in human skeletal muscle (Fig. 2G). This increase was primarily driven by a substantial post-RET increase in the protein abundance of the truncated isoform PGC-1α4, whereas PGC-1α1 protein abundance was unaltered (Fig. 2H and Supplemental Fig. 3). Since we have separated PGC-1α isoforms by molecular weight, it is possible that NT-PGC-1α, an additional truncated isoform of a similar projected molecular weight to PGC-1α4 (~40 kDa), also may have contributed to the increase in 40 kDa PGC-1α isoform protein abundance after RET. Although after acute RE the mRNA expression was increased for both PGC-1α1 and PGC-1α4, it is of interest that this only led to RET-induced increases in PGC-1α4 protein abundance. On the other hand, PGC-1α1 mRNA is increased after aerobic exercise^23^, resulting in elevated PGC-1α1 protein^35^. Moreover, while PGC-1α1 stimulates an oxidative phenotype, PGC-1α4 is not associated with large increases in oxidative metabolism^18,19^. Therefore, different exercise modes seem to drive divergent translational or post-translational processes, causing isoform-specific expression of PGC-1α protein in skeletal muscle.

The downstream metabolic effects of the aerobic exercise-induced increase in PGC-1α1 have been extensively studied. However, the transcriptional response induced by acute exercise and exercise training can be differentially regulated^36,37^, and little is known about the metabolic effect of increased PGC-1α4 protein in skeletal muscle. We investigated whether PGC-1α4 may regulate glycolytic metabolism. We found a significantly positive correlation between PGC-1α4 gene expression and multiple glycolytic transcripts in sedentary human skeletal muscle using RNAseq (Fig. 2I), indicating the potential role of PGC-1α4 in muscle glycolysis regulation. Likewise, only one glycolytic transcript was positively correlated with PGC-1α1 in sedentary human skeletal muscle (Fig. 2J). To further investigate this relationship, we analyzed glycolytic genes from myotubes overexpressing PGC-1α4 or PGC-1α1^19^. We found that PGC-1α4 overexpression increased 29 and decreased 10 glycolytic genes (Supplemental Fig. 4), whereas PGC-1α1 overexpression increased 22 and decreased 17 glycolytic genes in skeletal muscle (Supplemental Fig. 4), suggesting that PGC-1α4 is a more robust factor for increasing glycolytic gene expression than PGC-1α1 in skeletal muscle. It is important to keep in mind that only the 40 kDa truncated isoform of PGC-1α (including the PGC-1α4 isoform) protein, not PGC-1α1, is increased by RET. Therefore, even though increased protein expression of PGC-1α1 may also drive increases in glycolysis, it apparently does not do so following RET. Overall, our data strongly suggest that RE increases PGC-1α expression, which is positively correlated with glycolytic gene expression. Therefore, we sought direct evidence for the role of PGC-1α4 on glycolysis in muscle.

### Overexpressing PGC-1α4 in mouse myotubes enhances anaerobic glycolysis

**Fig. 3:**
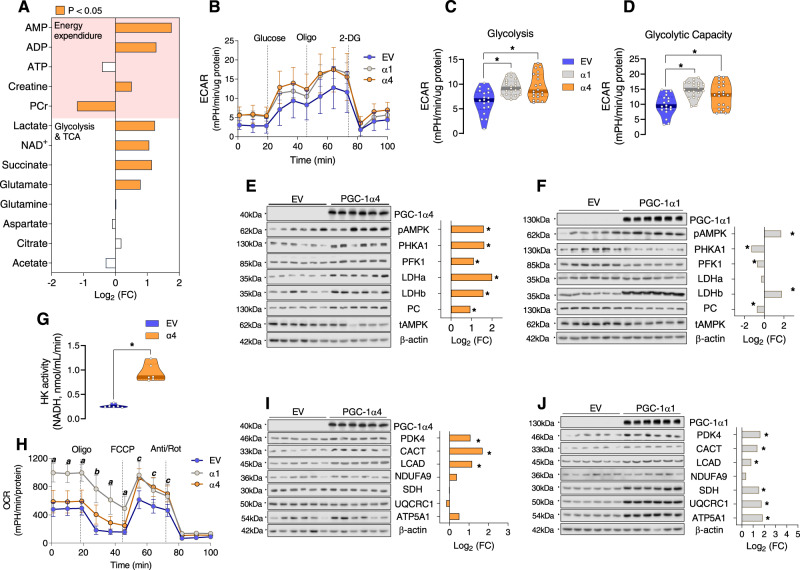
Overexpressing PGC-1α4 in mouse myotubes enhances glycolysis. **A** Bioenergetic metabolites were analyzed by NMR in myotubes transfected with an empty vector or an adenovirus overexpressing PGC-1α4 (*n* = 6 per group). Data are presented as log_2_ fold-change (FC) induced by PGC-1α4 overexpression. The orange colored bars represent a significantly changed value (*P* < 0.05). **B**–**D** Glycolysis was measured in myotubes following PGC-1α1 (α1) or PGC-1α4 (α4) overexpression using a Seahorse instrument. Values from α1 and α4 myotubes were compared to myotubes transfected with and empty vector (EV). Glucose, oligomycin (an inhibitor of oxidative phosphorylation which blocks ATP synthase), and 2-Deoxy-d-glucose (2-DG) were sequentially added to identify the change in extracellular acidification rate (ECAR). Glycolysis (measured in cells during exposure to glucose) and Glycolytic capacity (measured after exposure to oligomycin) were assessed by computing area under the curve using software of the Seahorse analyzer. One-way ANOVA was used with multiple comparisons. **E**, **F** The abundances of glycolysis-related proteins were analyzed following α4 and α1 overexpression in myotubes by immunoblotting. Unpaired two-tailed *t*-tests were used. **G** Hexokinase activity was measured in myotubes transfected with EV or α4. Unpaired two-tailed t-tests were used. **H** Oxygen consumption rate (OCR) was determined in myotubes overexpressing α4 or α1, and myotubes transfected with an EV by Seahorse analyzer. Oligomycin, FCCP (uncoupler), and antimycin/rotenone were sequentially added, and OCR was measured. At *P* < 0.05, “*a”* indicates that α1 > EV and α4; “*b*” indicates that α1 > EV and α4, but also α4 > EV; and “*c*” indicates that both α1 and α4 > EV. One-way ANOVA was used with multiple comparisons. **I**, **J** The abundance of fatty acid metabolism-related proteins and mitochondrial enzymes were analyzed following overexpression of α4 or α1 in myotubes by immunoblotting. Data are presented as log_2_ fold-change (FC) by PGC-1α isoform overexpression versus EV controls (*n* = 6 per group). Unpaired two-tailed *t*-tests were used. *P < 0.05 versus EV. Values are expressed as individual data points, Log_2_ FC, or mean ± SD. Significant labeled *P*-values in each panel from left to right are as follows: panel **C** = < 0.001 and <0.001; panel **D** = < 0.001 and 0.017; panel **G** = < 0.001.

To directly demonstrate the role of PGC-1α4 on glycolysis in muscle, we transfected adenovirus expressing PGC-1α4 in differentiated mouse myotubes. Using NMR spectroscopy, we found that PGC-1ɑ4 overexpression in myotubes increased energy expenditure (ATP utilization), evidenced by significantly higher AMP, ADP and creatine but lower phosphocreatine (PCr) levels (Fig. 3A). Glycolytic metabolites (lactate and NAD^+^) and TCA cycle intermediates (succinate and glutamate) were also significantly increased by PGC-1α4 overexpression in myotubes (Fig. 3A). Higher NAD^+^ and lactate are key indicators that PGC-1α4 enhances glycolysis. Oxidized NAD^+^ from NADH by lactate dehydrogenase-a (LDHa) is important to continue glycolysis. Indeed, we found that PGC-1ɑ4 overexpression increased glycolysis and glycolysis capacity in muscle cells (Fig. 3B–D). Similarly, PGC-1ɑ1 overexpression also resulted in enhanced glycolysis and glycolysis capacity (Fig. 3B–D). To further understand the molecular mechanisms for PGC-1ɑ4 and PGC-1α1 on cellular glycolysis, we assessed key proteins related to energy expenditure (AMPK phosphorylation), glycogenolysis (PHKA1), glycolysis (PFK1), and the lactate-pyruvate shuttle (LDHa and LDHb). We found that the expressions of all of these proteins were increased by PGC-1α4 overexpression (Fig. 3E). In contrast, although PGC-1α1 overexpression increased two of these factors (pAMPK and LDHb), the key glycolytic enzyme, PFK1, was decreased and others remained unchanged or decreased (Fig. 3F). Furthermore, hexokinase activity was robustly increased by PGC-1α4 overexpression (Fig. 3G). These results show that PGC-1α4 directly regulates anaerobic glycolysis via enhancing glycolytic pathway machinery and this effect is more robust than that of PGC-1α1.

Next, we measured oxygen consumption rate (OCR) in mouse myotubes overexpressing PGC-1ɑ4 or PGC-1ɑ1 and found that basal OCR was highest in myotubes overexpressing PGC-1α1, whereas no change in basal OCR was observed in myotubes overexpressing PGC-1α4 compared to empty vector (EV) (Fig. 3H), supporting the idea that PGC-1α4 increases anaerobic glycolysis. Oligomycin blocks ATP synthase and provides a measure of ATP-linked respiration. We found that ATP synthase was increased with PGC-1α1 overexpression (Fig. 3J). However, maximal oxygen consumption rate induced by FCCP (an uncoupler of oxidative phosphorylation) in both PGC-1α1 and PGC-1α4 myotubes was equally elevated after exposure to oligomycin (Fig. 3H), indicating a similar capacity of the electron transfer system with overexpression of both PGC-1α isoforms. In cells overexpressing PGC-1α1, oxygen consumption is not maximally stimulated (during FCCP) when ATP synthase is inhibited (by oligomycin) indicating that PGC-1α1 drives O_2_ consumption via the ETC. On the other hand, because cells overexpressing PGC-1α4 maximally elevated oxygen consumption while ATP synthase was blocked by oligomycin, this indicates that PGC-1α4 does not regulate the ETC. Further supporting this notion, we found that PGC-1α4 overexpression did not alter the abundance of key mitochondrial enzymes involved in the ETC including NDUFA9 (complex I), SDH (complex II), UQCRC1 (complex III), and ATP synthase (Fig. 3I). However, consistent with previous reports^38^, PGC-1ɑ1 overexpression increased key mitochondrial enzymes including SDH, UQCRC1, and ATP5A1 (Fig. 3J). These data suggest that PGC-1α1 could trigger enhanced aerobic glycolysis via increases in mitochondrial biogenesis, unlike PGC-1α4-which appears to induce increased anaerobic glycolysis.

A previous study has shown that both muscle glycogen and triglyceride provide energy during RE^39^. We found that both PGC-1ɑ4 and PGC-1ɑ1 overexpression increased the enzymes related to fatty acid oxidation including PDK4, CACT, and LCAD, indicating that both PGC-1ɑ isoforms are linked to the fat metabolism. We also found that although acute RE did not increase genes associated with fat metabolism and oxidative phosphorylation (Supplemental Fig. 5A), RET increased protein abundance of key fat metabolism enzymes (CD36 and CACT) in skeletal muscle similarly to AET (HIIT; high-intensity interval training) (Supplemental Fig. 5D–F). However, RET did not increase the protein abundance of electron transport chain enzymes, unlike HIIT (ETC) (Supplemental Fig. 5G–M). Additionally, acute RE or RET did not alter PDH activity (Supplemental Figs. 5B, C). Overall, these findings suggest that RET-induced PGC-1α4 expression supports fat metabolism, but not the ETC. Enhanced beta oxidation by RET is supported by elevation of mitochondrial beta oxidation proteins by RET (Supplement Fig. 5) concurrent to an increase in PGC-1α4 in muscle, thus providing fatty acid derived acetyl groups via acetyl CoA to the citric acid cycle.

Glycolysis and the TCA cycle tightly interact and share many metabolic pathways and intermediates. Therefore, we speculated that PGC-1α4 induced by RET could be involved in the interaction between glycolysis and TCA cycle. Pyruvate carboxylase (PC) catalyzes the carboxylation of pyruvate into oxaloacetate, which is essential for TCA cycle activation^40,41^. We found that PC protein abundance is increased with PGC-1ɑ4 overexpression (Fig. 3E), but not with PGC-1ɑ1 overexpression (Fig. 3F). Therefore, PGC-1α4 may help to support the TCA cycle via glycolysis and a PC-induced anaplerotic reaction, whereas PGC-1α1-driven increases in aerobic glycolysis likely occurs via highly activated oxidative phosphorylation. Overall, these data indicate that PGC-1α4 upregulates glycolysis by enhancing various enzymes for glucose catabolism. When glycolysis is elevated by PGC-1α4, glucose will need to be continuously supplied to the muscle.

### PGC-1α4 overexpression enhances glucose uptake in mouse myotubes

**Fig. 4:**
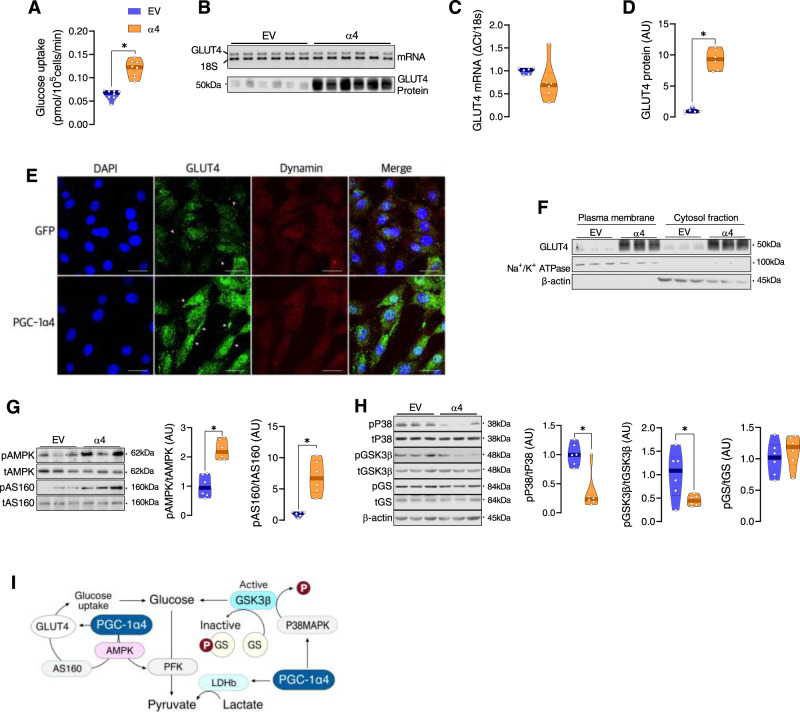
PGC-1α4 overexpression enhances cellular glucose uptake. **A** Glucose uptake was assessed using a bioluminescent kit assay in myotubes transfected with an empty vector (EV) or an adenovirus overexpressing PGC-1α4 (α4) (*n* = 8 per group). Unpaired two-tailed *t*-test was used. **B**–**D** GLUT4 mRNA was measured by semiquantitative RT-PCR, and GLUT4 protein content was measured by immunoblotting in myotubes expressing EV or overexpressing α4 (*n* = 6). Unpaired two-tailed *t*-tests were used. **E** GLUT4 antibody detection (green) and DAPI staining (blue) in cells transfected with EV or α4 show clustering of greater GLUT4 near the dynamin-labeled (red) cell membrane in α4 myoblasts. Experiments in panel **E** were repeated three times with similar results. White scale bars in each image are 30 µm in length. **F** GLUT4 was measured in the plasma membrane (indicated by the presence of Na^+^/K^+^ ATPase) and cytosolic (indicated by the presence of β-actin) fractions from myotubes transfected with EV or α4 (*n* = 3). **G**, **H** Representative immunoblots and quantification of phosphorylated AMPK, AS160, P38, GSK3β, and GS in cells transfected with EV or α4 are shown. Data are expressed as the phosphorylation of each signaling protein normalized to the abundance of its total protein content (*n* = 6 per group). Unpaired two-tailed *t*-tests were used. **I** Diagram of the hypothetical role of PGC-1α4 in glucose metabolism in muscle cells. PGC-1α4 enhances signaling and transport machinery to bring glucose into the cell, as well as the glycolytic enzymes necessary for metabolizing glucose. Further, inhibition of key signaling events involved in glycogen synthesis by PGC-1α4 suggest a favorable role of PGC-1α4 in glycolysis. *P < 0.05 versus EV. Values are expressed as individual data points. Significant labeled *P*-values in each panel from left to right are as follows: panel **A** = < 0.001; panel **D** = < 0.001; panel **G** = < 0.001 and <0.001; panel **H** = 0.002 and 0.027.

To test whether PGC-1α4 enhances glucose uptake, ensuring continuous glucose supply to muscle, we overexpressed PGC-1α4 in mouse myotubes, and found that PGC-1α4 increases glucose uptake (Fig. 4A). We observed that PGC-1α4 increases GLUT4 protein abundance without altering GLUT4 mRNA expression (Fig. 4B–D), suggesting that some post-transcriptional mechanism(s) drives the PGC-1α4-dependent increase in glucose transport machinery. Using immunofluorescent straining, we observed a higher amount of GLUT4 proximal to the plasma membrane in cells overexpressing PGC-1α4 (Fig. 4E). However, this finding does not necessarily mean that PGC-1α4 increases GLUT4 translocation per se, since we also observed in both the plasma membrane and cytosolic fractions that GLUT4 abundance was strikingly higher in cells overexpressing PGC-1α4 (Fig. 4F). Rather, the greater amount of GLUT4 in all regions of cells overexpressing PGC-1α4 ultimately results in a greater absolute amount of GLUT4 at the plasma membrane compared to EV cells, allowing for greater glucose transport. Some of the critical post-exercise signaling processes that are known to lead to greater glucose transport include phosphorylation of AMPK and Akt substrate of 160 kDa (AS160; TBC1D4)^26,42^. We observed significantly higher phosphorylation of AMPK and AS160 in PGC-1α4 overexpressed myotubes (Fig. 4G), suggesting PGC-1α4 increases GLUT4 translocation via enhanced signaling of AMPK and AS160. As PGC-1α4 is a transcriptional co-activator, its potential regulation of glucose transport and glycolysis likely occurs indirectly, potentially via activation of protein kinases induced by unknown factors. We discovered that PGC-1α4 overexpression decreases phosphorylation of P38-mitogen-activated protein kinase (P38MAPK) and glycogen synthase kinase β (GSK3β) (Fig. 4H), important signaling steps for controlling glycogen storage and glucose availability. Glycogen synthase (GS) phosphorylation (inactive form of GS) was unaltered by PGC-1α4 overexpression (Fig. 4H), but we observed enhanced GS phosphorylation after RET (Supplemental Fig. 6F), when PGC-1a4 abundance is high (Fig. 2H). We also found that MAPK and GSK3β phosphorylation were unaltered after either acute RE or RET (Supplemental Fig. 6A–D), and pGS was only increased with RET (not with acute RE) (Supplemental Fig. 6E, F). A previous study has shown that phosphorylation of GS is increased when glycogen is “super-compensated” above normal resting levels after muscle contractions in rabbit^43^. These results suggest PGC-1α4 acts via P38MAPK, GSK3β and GS to regulate glycogen synthesis and glycolysis in muscle (Fig. 4I). However, to clearly understand the influence of PGC-1α4 on this signaling pathway in vivo, further investigation is required since this co-activator cannot by itself regulate these metabolic processes.

### Resistance exercise training-induced PGC-1ɑ4 cooperates with PPARβ to regulate glycolysis

**Fig. 5:**
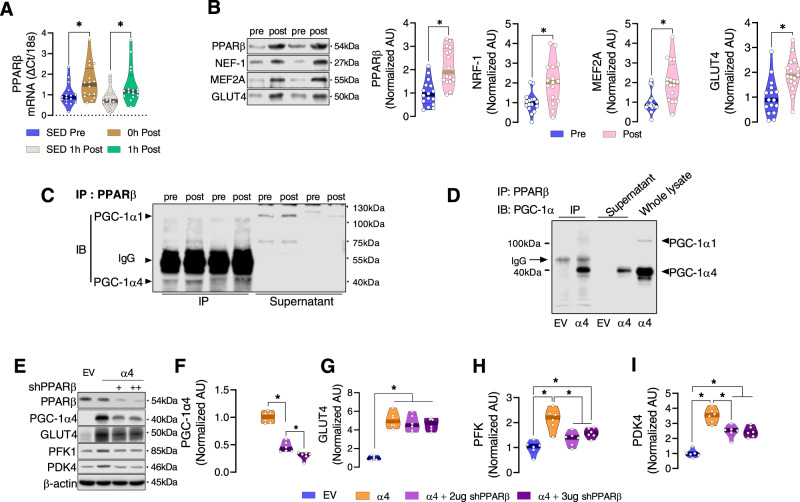
Resistance exercise training-induced PGC-1ɑ4 cooperates with PPARβ to regulate glycolysis. **A** PPARβ mRNA was determined by qPCR in muscle biopsy samples of healthy participants before (SED Pre), within 10 min after (0 h Post), and 1 h after (1 h Post) a one-legged resistance exercise (RE) bout (*n* = 16). Time-matched control muscle samples were obtained from the non-exercised leg at 1 h post-RE (SED 1 h Post) (*n* = 16 per group). One-way ANOVA was used with multiple comparisons. **B** Protein abundance of PPARβ and its downstream related proteins was determined in muscle before (Pre) and after (Post) RET. Representative immunoblots for each protein and quantification of the relative change after training for PPARβ, NRF-1, MEF2A, and GLUT4 are displayed (*n* = 16). Paired two-tailed *t*-tests were used. **C** PPARβ was immunoprecipitated from pooled muscle samples before (pre) and after (post) RET, then the immunoprecipitate was immunoblotted for PGC-1α1 and PGC-1α4 to determine binding between PPARβ with PGC-1α1 and PGC-1α4. Owing to the large amount of protein required to immunoprecipitate enough PPARβ for immunoblot detection of PGC-1α1 and PGC-1α4, samples of multiple participants before and after training were pooled together, resulting in a smaller *n* for PGC-1α isoform quantification (*n* = 2). **D** The binding between PPARβ and PGC-1α isoforms was confirmed using cells overexpressing PGC-1α4. Experiment shown in panel **D** was performed once. **E**–**I** PPARβ was silenced by shPPARβ overexpression in myotubes, then PGC-1α4 was overexpressed (*n* = 6). The abundance of some of the important glycolytic proteins (GLUT4, PFK1, and PDK4) was determined by immunoblotting. One-way ANOVAs were used with multiple comparisons. *P < 0.05. Values are expressed as individual data points. Significant labeled *P*-values in each panel from left to right are as follows: panel **A** = 0.024 and 0.020; panel **B** = < 0.001, 0.002, <0.001, <0.001; panel **F** = < 0.001 and 0.010; panel **G** = < 0.001; panel **H** = < 0.001, <0.001, and <0.018; panel **I** = < 0.001, <0.001, and 0.002.

Since PGC-1α4 is a co-activator, it is required to interact with a nuclear receptor to drive the transcription of its target genes. Peroxisome proliferator-activated receptor β (PPARβ) is a dominant nuclear receptor in skeletal muscle^25,44,45^. We identified that acute RE rapidly increases PPARβ gene expression (Fig. 5A) and RET increases PPARβ protein abundance in skeletal muscle (Fig. 5B). Furthermore, the protein expression of downstream targets of PPARβ, nuclear respiratory factor 1(NRF1), myocyte enhancer factor 2 (MEF2), and GLUT4 are all significantly increased in skeletal muscle by RET (Fig. 5B). We tested whether RET increases binding of truncated PGC-1α (which includes PGC-1α4) to the nuclear receptor PPARβ in human skeletal muscle (Fig. 5C). We found that the interaction between PGC-1α4 and PPARβ was significantly higher after RET (Fig. 5C), even though the RET samples were taken at 72 h after last bout of exercise. However, a similar interaction between PGC-1α1 and PPARβ was not observed (Fig. 5C). We also detected greater PGC-1α4, but not PGC-1α1, protein content after RET (Fig. 2H) suggesting that increased binding of PGC-1α4 to PPARβ is due to either (1) an increased affinity of these two proteins with each other or (2) an increased availability of both proteins. We also confirmed the binding of PGC-1α4 to PPARβ using myotubes overexpressing PGC-1α4 (Fig. 5D). As we detected increased glycolysis-related proteins in myotubes overexpressing PGC-1α4 (Fig. 3E), we tested if PPARβ is critical for the increased expression of some of the important proteins involved in glucose uptake and glycolysis. We found that in myotubes which overexpress PGC-1α4, when PPARβ was silenced there is a blunted effect on the abundance of glycolytic proteins GLUT4, PFK, and PDK4 (Fig. 5E–I). Interestingly, PGC-1α4 was decreased when PPARβ was silenced (Fig. 5F). Previous studies have shown that PPARβ increases PGC-1α protein stability^46,47^ by binding to PGC-1α and limiting ubiquitination to block degradation^46^. Thus, the lower abundance of key glucose metabolism proteins PFK and PDK by PPARβ silencing is likely a result of poor binding of PPARβ with PGC-1α4 to regulate glycolytic gene expression. Collectively, these results support the hypothesis that enhanced glycolysis following RET is facilitated by increased PGC-1α4 and PPARβ protein abundance, as well as binding of PGC-1α4 (and potentially also NT-PGC-1α) with PPARβ. However, following a single bout of RE we were unable to detect changes in protein abundance of PGC-1α4 (Supplemental Fig. 2), PPARβ (Supplemental Fig. 7), or binding of PGC-1α4 to PPARβ (Supplemental Fig. 8). Therefore, it is likely that the interaction between PPARβ and PGC-1α4 that occurs after 12 weeks of RET does not explain the increased glycolysis observed in skeletal muscle after an acute bout of RE.

### The influence of PGC-1ɑ4 on muscle glycolysis is AMPK dependent

**Fig. 6:**
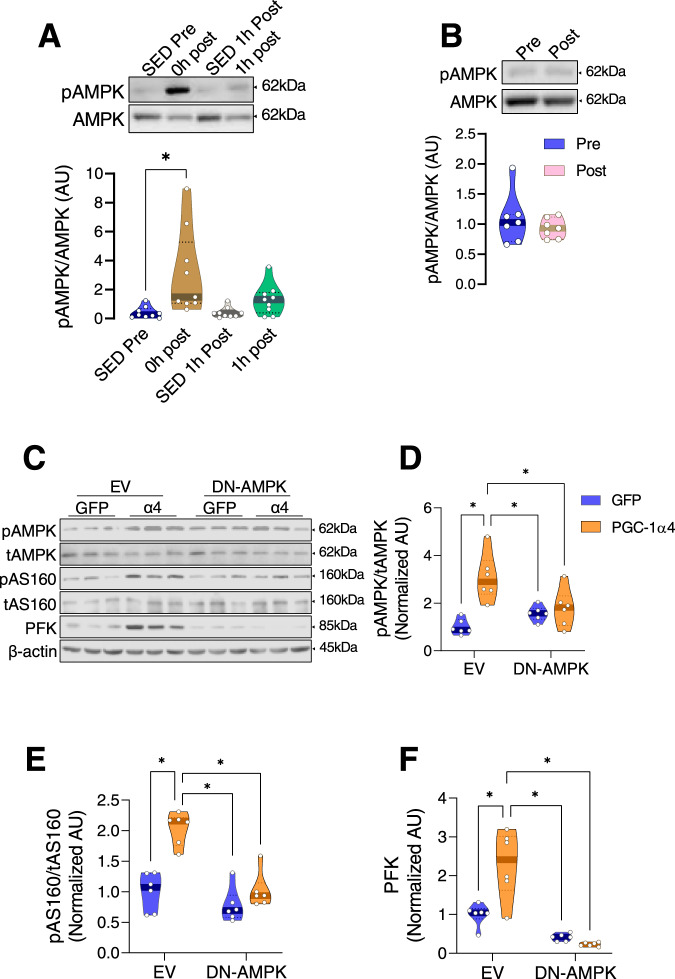
The influence of PGC-1ɑ4 on muscle glycolysis is AMPK dependent. **A**, **B** Representative immunoblots and quantification of phosphorylated AMPK in human muscle obtained after an acute resistance exercise bout (**A**) or after RET (**B**). One-way ANOVA was used with multiple comparisons for panel **A**, and paired two-tailed *t*-test was used for panel **B**. Data are expressed as the phosphorylation of each signaling protein normalized to the abundance of its total protein content (*n* = 9 for acute RE, *n* = 7 for RET). **C**–**F** Representative immunoblots and quantification of AMPK, AS160, and PFK in cells transfected with GFP or PGC-1α4 after EV or DN-AMPK transduction are shown. Data are expressed as the phosphorylation of each signaling protein normalized to the abundance of its total protein content and PFK was normalized with β-actin (*n* = 6 per group). One-way ANOVAs were used with multiple comparisons *P < 0.05 versus empty vector (EV). Values are expressed as individual data points. Significant labeled *P*-values in each panel from left to right are as follows: panel **A** = 0.013; panel **D** = < 0.001, 0.005, and 0.033; panel **E** = < 0.001, <0.001, and <0.001; panel **F** = 0.006, <0.001, and <0.001.

We next sought to identify if AMPK, a signaling protein highly known for its post-exercise role in mobilizing fuels from energy stores^26^, interacts with PGC-1α4 after an acute bout of RE. AMPK is a sensor of cellular metabolic alterations that can communicate with downstream effectors that can elicit responses to stressful metabolic stimuli. During acute RE, ATP is rapidly used, which increases the cellular ratio of AMP/ATP (and ADP/ATP), causing activation of AMPK. We found that immediately after RE, when AMP levels are high, that AMPK phosphorylation (pAMPK) was increased, which was almost completely back to basal levels by 1 h post-RE (Fig. 6A). Consistent with previous reports, pAMPK was unaltered after RET (Fig. 6B). This is expected since the muscle biopsies were obtained 72 h after the last bout of exercise. To identify whether PGC-1α4 cooperates with AMPK, dominant-negative-AMPK (DN-AMPK) was overexpressed in mouse myotubes, blocking AMPK activation. This was followed by PGC-1α4 overexpression. We found that PGC-1α4 overexpression increased AMPK phosphorylation, and that pAMPK was completely blunted with DN-AMPK (Fig. 6C, D). Moreover, the increase in both AS160 phosphorylation and PFK abundance that is observed with PGC-1α4 overexpression was also completely blunted when AMPK could not be phosphorylated (Fig. 6C, E, F). Phosphorylation of AS160 by increased AMPK activation following muscle contraction regulates GLUT4 translocation to the plasma membrane in skeletal muscle^48^. PFK can promote glycolysis rate during muscle contraction^49,50^. Our findings support the notion that PGC-1α4 promotes glucose uptake and glycolysis in an AMPK activation-dependent manner. Of course, this does not rule out the possibility that other acute exercise-related signaling proteins may also interact with PGC-1α4.

## Discussion

The current study provides substantial experimental evidence supporting the hypothesis that PGC-1α4 is a key regulator of energy supply via glycolysis, an oxygen-independent metabolic pathway for rapidly providing ATP^22^ to perform quick and powerful muscle contractions during RE. We demonstrated that RET increased the protein expression of the 40 kDa truncated isoform of the transcriptional co-activator PGC-1α, which includes PGC-1α4. We also showed that PGC-1α4 promotes the cellular anaerobic glycolytic pathway in a PPARβ- and AMPK-dependent manner. Elucidation of these molecular pathways unfolds potential opportunities for therapeutic discovery to ameliorate not only sarcopenia and related frailty but also cardiometabolic complications in aging humans and in type 2 diabetes.

In addition to demonstrating that activation of the glycolytic pathway occurs concurrent with increased PGC-1α4 (and/or NT-PGC-1α) protein expression following RET in humans, we performed muscle cell-based studies to directly demonstrate that PGC-1α4 enhances glycolysis and glycolytic capacity. Like in human muscle following RET, PGC-1α4 in muscle cell lines enhanced the activity of hexokinase concurrent with an increase in glycolysis and glycolytic proteins. Based on NMR measurements of bioenergetic metabolites (increased ADP and AMP; and decreased PCr and ATP), we demonstrated that energy utilization is high when PGC-1α4 is overexpressed in muscle cells. However, unlike the higher O_2_ consumption that is observed when PGC-1α1 is overexpressed, PGC-1α4 overexpression did not increase O_2_ consumption. Together, these data illustrate that PGC-1α4 increases the anaerobic glycolytic pathway to meet ATP needs during RE. Owing to the high energy demand with muscle contractions during RE, large amounts of ATP need to be rapidly provided to the contracting muscle. PCr and anaerobic glycolysis can produce ATP much faster than oxidative phosphorylation, therefore anaerobic glycolysis is a preferred metabolic pathway of ATP generation for contracting muscle during RE^22^.

RE has been shown to elicit specific metabolic and molecular responses in skeletal muscle resulting in specific functional outcomes^51,52^. For example, clinical studies have shown that RET improves glucose and lipid disposal in T2DM and in elderly people^7–12^. The most critical enzymes in the glycolytic pathway are HK, PHKA, and PFK, and the regulation of these enzymes is tightly controlled^53^. Impaired glycolysis occurs in skeletal muscle with aging^54^ and substantive reduction in oxidative glycolysis in T2DM is not compensated for by an adequate increase in non-oxidative glycolysis^55^. These reported impairments in glycolysis are likely due to disrupted regulation of glycolytic enzymes such as HK, PFK, Aldolase, and PK. For example, lower HKII activity has been observed in patients with T2DM^56^. We observed high activity of HK in human skeletal muscle after RET, which could be involved in glucose uptake by preventing the outflow of glucose from the cell. We also showed that the activity of PFK was rapidly increased in human skeletal muscle after a single bout of RE. Further, the enhanced peripheral (mostly muscle) glucose uptake that is reported after RET in humans^18^ is associated with enhanced HK activity and transcripts of the glycolytic pathway. Together, these data from human and cell-based studies support the notion that the glycolytic pathway is activated by RE and RET and appears to at least be partially regulated by PGC-1α4. However, since PGC-1α4 is a transcriptional co-activator, its protein expression alone cannot explain the RET-induced metabolic effects, and it must interact with a nuclear receptor.

Here, we provide evidence that PGC-1α4 cooperates with PPARβ, which is an abundant nuclear receptor in skeletal muscle that binds to and prevents the degradation of total PGC-1α after AET^46^. However, the interactive role between PPARβ and PGC-1α4 after RET was not known. We demonstrated that RET increased binding of the 40 kDa truncated form of PGC-1α to PPARβ in human skeletal muscle. As stated earlier, the truncated isoforms of PGC-1α, including both PGC-1α4 and NT-PGC-1α, are observed at the same size of 40 kDa. Thus, our human experiments cannot distinguish if PPARβ is increasing post-RET binding to PGC-1α4, NT-PGC-1α, or both PGC-1α4 and NT-PGC-1α. Therefore, to directly test the importance of PPARβ in mediating the effects of PGC-1α4, we silenced PPARβ in mouse myotubes overexpressing PGC-1α4, which resulted in the downregulation of PGC-1α4 abundance, supporting the role of PPARβ in regulating the protein abundance of PGC-1α4. Moreover, PFK and PDK abundance which are enhanced by PGC-1α4, decreased when PPARβ was silenced in myotubes overexpressing PGC-1α4. Therefore, these myotube experiments suggest that the 40 kDa isoform of PGC-1α, which shows increased binding to PPARβ following RET in human muscle, is likely the PGC-1α4 isoform. However, we still cannot definitively rule out the possibility that NT-PGC-1α also contributes to this observation in humans. Furthermore, RET increased some of the key downstream PPARβ targets NRF1, MEF2, and GLUT4, suggesting increased PGC-1α4 binding to PPARβ with RET enhances PPARβ’s transcriptional regulation. Together, these results indicate that enhanced PGC-1α4, which occurs following RET, promotes glycolytic rate in a PPARβ dependent manner. Thus, increased expression of PPARβ with RET may mediate the PGC-1α4 effects on glycolysis.

However, the increased protein abundances of PGC-1α4 and PPARβ after RET cannot explain the increased glycolysis after an acute bout of RE as we were unable to detect increased PPARβ and PGC-1α4 binding after a single bout of RE in skeletal muscle. Therefore, some other mechanism(s) must exist which enhance glycolysis after a single bout of RE. It seemed logical that the “energy sensor” protein AMPK, which is known to be regulated by exercise, may also interact with PGC-1α4 to regulate glycolysis after acute RE. As expected, we found that pAMPK was enhanced in human skeletal muscle following acute RE but the increased abundances of PFK (a key glycolytic enzyme enhanced by acute RE) and AS160 (a regulator of GLUT4 translocation) in myotubes overexpressing PGC-1α4 were completely blunted when AMPK activation was prevented. These results support the idea that the interaction of PGC-1α4 with activated AMPK is needed to exert its effect on glucose uptake and glycolysis. Of interest, previous studies have shown that PPARβ cooperates with AMPK to regulate GLUT4 protein expression and glucose uptake^27^, as well as increase glucose oxidation^57^. It is well known that AMPK activation by acute exercise is returned to baseline within hours after exercise^58–61^. Moreover, the training effect of resistance exercise does not affect basal AMPK activity as noted when we analyzed muscle samples collected 72 h following last bout of RE in participants that underwent three months of RET (Fig. 6B), unlike the effect of an acute bout of RE (Fig. 6A). Therefore, AMPK’s role in enhanced post-exercise glycolysis appears to primarily occur during and subsequent to an acute exercise stimulus. The current study has revealed that while the PGC-1α4-AMPK interaction may be critical for enhanced glycolysis and glucose uptake immediately following RE, the PGC-1α4-PPARβ interaction plays a critical role in sustaining enhanced muscle glycolysis and glucose uptake following RET.

PGC-1α4 is a spliced variant of the transcriptional co-activator PGC-1α1 and there is strong evidence that PGC-1α1 plays a key role in AET-induced improvements in glucose metabolism, likely through the PGC-1α1-mediated regulation of mitochondrial biogenesis. Although RET has only minimal impact on mitochondrial function^18^ and does not alter PGC-1α1 protein, glucose metabolism is equally improved following RET compared to AET, but by a distinct mechanism(s). A previous report demonstrated that PGC-1α4 regulates RET-induced muscle hypertrophy^19^. In addition, the current study demonstrated that protein expression of the 40 kDa truncated form of PGC-1α (which includes PGC-1α4) is increased after RET and plays a crucial role in the energy metabolism involving glucose and fat, especially in activating the anaerobic glycolytic pathway for anaerobic glycolysis and ATP production. These divergent but complementary pathways resulting from two specific exercise programs have beneficial effects on fuel metabolism. A strong case can be made for PGC-1α4 as a potential target for therapeutic research to improve insulin sensitivity, and prevent diabetes and cardiometabolic diseases. Based on previously published findings^19^, PGC-1α4 may also be a potential target for enhancing muscle hypertrophy, thus preventing sarcopenia and related frailty of aging.

In conclusion, our human and cell line studies together reveal that the underpinning mechanisms of RET-induced metabolic benefits appears to at least be partially regulated by PGC-1α4, which regulates glycolytic gene expression and promotes anaerobic glycolysis and glucose uptake in a PPARβ-dependent manner. Meanwhile, the acute effect of RE is AMPK-dependent. The discovery of these pathways may lead to future translational studies in preventing and treating multiple aging-related and cardiometabolic disorders.

## Methods

### Human exercise experiments

All studies were approved by the Mayo Clinic Institutional Review Board and all participants gave their informed consent to participate in the studies. The study design and conduct complied with all relevant regulations regarding the use of human study participants and was conducted in accordance with the criteria set by the Declaration of Helsinki.

#### Acute resistance exercise study

Eight male and 9 female healthy participants were recruited between 18-55 years of age with a BMI between 21.0 and 32.0 kg/m^2^. Participant characteristics are presented in Supplemental Table 1. On their initial visit, participants performed a one-repetition maximum (1-RM) exercise test using a one-legged knee-extension machine. The 1-RM test began with a 5 rep warmup at 40lbs. Participants then began performing 1 repetition every 60–90 s with progressively increasing weight until the participant could no longer lift the weight. Weight was increased with each repetition at self-selected increments of no more than 20lbs. If a weight could not be lifted after two attempts, then the last successfully lifted weight was determined to be the participant’s 1-RM. Participants returned 2–30 days after the 1-RM test (mean = 13 days) after an overnight fast at 0700. A percutaneous needle muscle biopsy was obtained under local anesthesia (2% lidocaine buffered with sodium bicarbonate) from the vastus lateralis (VL) of one leg (Sedentary Leg) using a modified Bergstrom muscle biopsy needle. Participants then performed 3 sets of 10 repetitions at 70% of their 1-RM of one-legged knee-extension exercise on the contralateral leg (Exercised Leg)^62,63^. Immediately post-exercise (0 h Post; within ~5–10 min from the last set of exercise) a second muscle biopsy was obtained from the VL of the exercised leg. Approximately 1-h post-exercise (1 h Post) two more muscle biopsies were obtained, one biopsy from each leg. After each biopsy, samples were quickly dried of excess blood, and any visible adipose and/or connective tissue was removed at ~0–4 °C. Samples were then frozen in liquid nitrogen and stored at –80 °C until analysis.

#### Exercise-training study

Supervised resistance (RT) or aerobic (HIIT; high-intensity interval training) exercise-training programs were conducted at the Dan Abraham Healthy Living Center for 3-months in healthy young and older participants. Data presented from exercise-trained participants were obtained from samples that were collected as a part of a previously published project^18^. Owing to the limited remaining tissue samples from the previously published project, samples from a subset of these participants were used in the current analysis. Detailed experimental procedures, including the complete exercise list, participants characteristics, and training differences in aerobic fitness, muscle mass, strength, and insulin sensitivity can be found in our previous publication^18^. Muscle biopsies were obtained in the morning following an overnight fast at 1000 h from the VL of participants before and after 3-months of exercise training. Post-training muscle biopsies were obtained at least 72 h after the last exercise bout.

### Cell culture

Cell culture was performed as previously described^64^. C_2_C_12_ mouse muscle cells (source from ATCC, cat. no. CRL-1772, female) were obtained from ATCC (Manassas, VA). Cells were maintained in 5% CO_2_ at 37 °C and grown in DMEM (Sigma-Aldrich, cat. no. D-5796, St. Louis, MO) containing 10% fetal bovine serum (Sigma-Aldrich, cat. no. F-2442), penicillin (100 U/ml), and streptomycin (100 µg/ml) (Thermo Fisher Scientific, cat. no, 15140122, Waltham, MA). C_2_C_12_ cells were differentiated into myotubes by replacing the media with new media containing 2% horse serum (Thermo Fisher Scientific, cat. no. 26050070), penicillin (100 U/ml), and streptomycin (100 µg/ml) (Thermo Fisher Scientific, cat. no. 15140122).

### Adenoviral PGC-1ɑ1 or PGC-1ɑ4 vector preparation

pAd-CMV vector containing PGC-1ɑ1 or PGC-1ɑ4 were gifted by Bruce M. Spiegelman (Harvard Medical School). Amplified and purified plasmid DNA was digested with Pac I to expose its inverted terminal repeats (ITR), and then was used to transfect HEK293 cells to produce adenoviral PGC-1ɑ1 or PGC-1ɑ4 vectors. Adenoviral shPPARβ, Dominant-negative AMPK (DN-AMPK), and green fluorescent protein (GFP) were provided from John O Holloszy (Washington University School of Medicine). Each adenoviral vector or GFP (as control empty vector (EV)) were applied to differentiated myotubes.

### Glucose uptake in mouse myotubes

We used a commercially available glucose uptake kit (Promega, cat. no. J1341, USA) following the manufacturer’s directions. Briefly, after mouse myotubes were fasted overnight, the medium was replaced with the new medium without serum and incubated at 37 °C in a cell incubator. After 1 h, the medium was replaced with PBS containing 0.1 mM 2DG followed by incubation for 30 min at room temperature. Stop buffer and neutralization buffer were then sequentially added. 2DG6P detection reagent was then added followed by a 1 h incubation at room temperature. Luminescence was detected using a microplate reader.

### Gene expression analysis

mRNA expression was determined from the exercise-training study and from cell culture studies as previously described^18^ with slight modifications. In brief, total RNA was isolated from biopsies collected from the exercise-training study, quality assessed using RNA Integrity Number (RIN), and sequencing libraries were prepared with TruSeq RNA Sample Prep Kit v2. Libraries were sequenced on a HiSeq 2000 sequencer using TruSeq SBS sequencing kit version 3 and HCS version 2.0.12.0 software. Sequenced read data was processed using previously published MAP-RSeq pipeline^65^ configured to use hg38 genome. Genes and corresponding read counts observed in each sample were extracted and processed using edgeR software (version 3.22.5)^66^ for differential expression. Owing to the paired nature of the study (i.e., post vs. pre), a negative binomial generalized log-linear model blocked for subjects was utilized to model gene expression data and infer differential expression *P*-value associated with training time as contrast (i.e., post vs. pre). Genes with a FDR-corrected *P*-value of <0.05 and an absolute log_2_ fold-change of >0.5 (where 0.0 signifies no change) were considered for further analysis. Independently, qPCR was used for all measured transcripts from the acute resistance exercise study and for all PGC-1α isoform transcripts from the exercise-training study. For the exercise-training study, PGC-1α isoforms were measured in 35 participants before exercise training so that correlations could be run between PGC-1α isoforms and glycolytic genes (see Fig. 2I, J). Total RNA was isolated using TRIzol reagent (Thermo Fisher Scientific, Houston, TX). After total RNA was extracted from muscle samples and cDNA was synthesized using the SuperScript IV First-Strand Synthesis System for RT-PCR (Thermo Fisher Scientific). Quantitative RT-PCR was performed in 384-well clear plates with 10 μl reaction volume using SYBR Green (Applied Biosystems, Foster City, CA, USA). Amplification conditions were 10 min at 60 °C followed by 40 cycles of denaturing (95 °C for 15 s) and annealing (60 °C for 60 s) using a ViiA7 thermocycler (Applied Biosystems). Gene-specific primers are shown in supplemental table 2. mRNA expression values were quantified by the 2ΔΔCt method, whereby ΔCt = 18S Ct – target gene Ct, resulting in data being presented in arbitrary units (AU). 18s was chosen as the reference mRNA after comparison of 18s, Gaphd, and Actb expressions within samples using the ΔCt approach^67^ showed the expression stability was the greatest in 18s. For gene expression analysis in the acutely exercise study, a subset of 16 participants (8 male and 8 female) were used due to sample limitations of one of the female participants. The demographics (age, BMI, and recorded 1-RM) of this cohort are not significantly different from our larger cohort of 17 participants (supplemental table 1). For the resistance trained samples a total of 8–14 biopsies per group were used due to remaining sample limitations.

### Semiquantitative RT-PCR

GLUT4 mRNA was determined as previously described^27^. Total RNA from mouse myotubes were isolated using TRIzol reagent (Thermo Fisher Scientific, Houston, TX). Total RNA was reverse transcribed into cDNA by using random primer and Im Prom-II Reverse Transcriptase (Promega, Madison, WI). Each cDNA sample was added to a PCR master mix (Promega) mixture, and 10 pmol of both sense and antisense primers of GLUT4 (forward 5 = -TGGAGCTCGATGACAGTGAC-3 = , reverse 5 = -GTACTGGCTGTCAGGGTGGT-3 = ); (forward 5 = -ctcaaccaactggcca 3 = , reverse 5 = -cagctcctatggtggcgtag-3 = ). To perform PCR amplification, after the lid was warmed at 94 °C for 2 min, the PCR mixtures were subjected to a 27-cycle profile, and 18S rRNA expression was simultaneously measured as an internal standard by using a QuantumRNA 18S Internal Standard Kit (Ambion, Austin, TX). PCR products were separated by electrophoresis on 1.5–2% agarose, stained with SYBR Green (Molecular Probes, Eugene, OR), photographed, and analyzed by densitometry.

### Immunoblotting

Western blotting was conducted as previously described^64^. Briefly, frozen muscles were powdered and then homogenized in a 15:1 (vol/wt) ratio of ice-cold RIPA buffer supplemented with protease inhibitor cocktail (Thermo Fisher Scientific, Houston, TX). The homogenates were then centrifuged at 1000 × *g* for 15 min at 4 °C, and the supernatant was used. Proteins were separated by SDS-PAGE and transferred to nitrocellulose membranes, then blocked for 1 h in 5% w/v skim-milk in TBS-T (tris-buffered saline (TBS) containing 0.1% tween 20 (Sigma-aldrich #P1379, St. Louis, MO) and 0.2% imidazole (Sigma-aldrich #56750)). Membranes were incubated overnight at 4 °C with primary antibodies at dilutions of 1:1000 in 2% w/v bovine serum albumin (BSA, Sigma-Aldrich #A7906, St. Louis, MO) in TBS-T. The antibody used for detection of PGC-1ɑ1 and ɑ4 was gifted by Thomas Gettys (Pennington Biomedical Research Center)^68^, which can now be found as PGC-1ɑ (cat. no. 516557) from Millipore (Billerica, MA). Following immunoprecipitation, immunoblotting with the PGC-1α antibody allows for the determination of different PGC-1α isoforms based on molecular weight (see Fig. 5C). Antibodies used for specific detection of MEF2A (cat. no. 9736), AMPKα (cat. no. 2793), α-tubulin (cat. no. 2125), phospho-AMPKα Thr-172 (cat. no. 2531), phospho-AS160 (cat. no. 4288), AS160 (cat. no. 2447), phospho-Glycogen Synthase (cat. no. 3891), Glycogen Synthase (cat. no. 3893), phospho-GSK3β (cat. no. 5558), GSK3β (cat. no. 12456), p38 MAPK (cat. no. 8690), and phospho- p38 MAPK (cat. no. 4511) were from Cell Signaling Technologies (Danvers, MA); PPARβ (cat. no. PA5-29678), NDUFA9 (cat. no. 459100), COX4 (cat. no. A21348), UQCRC1 (cat. no. 459140), and ATP5A1 (cat. no. 459240) were from Thermo Fisher Scientific (Houston, TX); PFK (cat. no. SC-166722), PDK4 (cat. no. SC-130841), SOD2 (cat. no. sc-137254), Catalase (cat. no. sc-271803), and NRF-1 (cat. no. SC-23624) were from Santa Cruz Biotechnology (Santa Cruz, CA); β-actin (cat. no. A5441) was from Sigma Aldrich; GLUT4 (cat. no. ab-654), Na^+^/K^+^ ATPase (cat. no. ab76020), LCAD (cat. no. 196655), and UQCRC2 (cat. no. ab14745) were from Abcam (Cambridge, MA); CD36 (cat. No. 18836-1-AP), LDHa (cat. no. 19987-1-AP), LDHb (cat. no. 14824-1-AP), Pyruvate Carboxylase (PC; cat. no. 16588-1-AP), and CACT (cat. no. 19363-1-AP) were from proteintech (Rosemont, IL); Succinate dehydrogenase (SDH; cat. no. 439300) was from Innovative Research; Cyt C (cat. no. 556433) was from BD Biosciences; PHKA1 (cat. no. GTX109401) was from GeneTex (Alton, CA); CPT1M (cat. no. CPT1M11-A) from Alpha diagnostic (San Antonio, TX); and secondary antibodies such as donkey anti-mouse (cat. no. 715-035-150), donkey anti-rabbit (cat. no. 711-035-152), and streptavidin (cat. no. 016-030-084) were obtained from Jackson Immunoresearch Laboratories (West Grove, PA). Secondary antibodies were used at a dilution of 1:10,000. Antibody-bound protein was detected by Clarity Western ECL Substrate (Bio-Rad, cat. no. 170-5060, Hercules, CA). Signal was visualized using a C-DiGit blot scanner (Li-COR Bioscience, cat. no. 3600-00, Lincoln, NE). Immunoblotting of AMPK in human muscle was performed on 9 acutely exercised and 7 exercise-trained samples due to sample limitations. The blots were then incubated for 1 h at room temperature with the appropriate horseradish-conjugated secondary antibody in diluted TBS-T containing 2% w/v skim-milk. Secondary antibody such as donkey anti-mouse (#715-035-150) and donkey anti-rabbit (#711-035-152) were obtained from Jackson Immunoresearch Laboratories (West Grove, PA). Antibody-bound protein was detected by Clarity Western ECL Substrate (Bio-Rad #170-5060, Hercules, CA). β-actin was used as a loading control, and membranes were stripped before or after probing for β-actin for the detection of other proteins of interest. β-actin is unaltered by exercise training and is acceptable as a loading control (Supplemental Fig. 9).

### Extraction of plasma membrane protein

We used a commercially available plasma membrane protein extraction kit (BioVision, Milpitas, CA) following the manufacturer’s directions. Briefly, cells in a T75 flask were collected and then spun down. Cells were washed with PBS and cell membranes were broken by freeze thawing, and then samples were centrifuged at 700 × *g* for 10 min at 4 °C and the supernatant was collected. The supernatant was centrifuged at 10,000 × *g* for 30 min at 4 °C and the pellet was collected. The pellet was resuspended in 200 µl of the upper phase solution, and then 200 µl of lower phase solution was added and was mixed well, followed by incubation on ice for 5 min. Another fresh phase tube without sample was prepared with 200 µl of upper phase solution and 200 µl of lower phase solution followed by centrifugation 1000 × *g* for 5 min. An upper phase was collected and then was diluted with 5 volumes of water followed by incubation for 5 min on ice. Samples were spun at 15,000 × *g* for 10 min at 4 °C and then the pellet (which contained isolated plasma membrane proteins) was used for analysis.

### Immunoprecipitation

In all, 10 µl PGC-1ɑ or PPARβ antibodies were rotated with PureProteome™ Protein A/G Mix Magnetic Beads (Millipore; LSKMAGAG10) for 1.5 h at room temperature. A total of 250 µg (for cells) or 700 µg (for muscle tissue) of protein from homogenized samples of each group was added in the target antibody/magnetic bead mixture and then rotated overnight at 4 °C. The antibody-antigen complexes captured on magnetic beads were probed using immunoblot analysis for PGC-1ɑ1 and PGC-1ɑ4.

### Nuclear magnetic resonance (NMR)

NMR was conducted as previously described^64^. Briefly, cells were treated with 200 µl of 6% HClO_4_ and ground for 30 s with a hand homogenizer. The mixture was vortexed and frozen in liquid nitrogen. The samples were thawed and spun down at 10,000 × *g* for 15 min. The supernatant was collected, and the pellet was re-extracted with 100 µl of 6% HClO_4_. Combined extracts were neutralized with 105 µl of 2 M KHCO_3_. The mixture was spun down at 10,000 × *g* for 15 min and supernatant (300 µl) was collected. Then 200 µL of phosphate buffer (pH 7.4) and 50 µl of TSP-*d*_4_ solution in D_2_O (1 mM) were added, and the sample was transferred to a 5 mm NMR tube.

NMR spectra were acquired on a Bruker 600 MHz Avance III HD spectrometer (Bruker, Billerica, MA), using 1D NOESY pulse sequence with presaturation (noesygppr1d). The spectra were analyzed using Chenomx NMR suite software (Chenomx, Edmonton, Canada). Metabolite concentrations were exported as μM in the NMR sample and recalculated as nmoles in the cell extract.

### Glycolysis measurement and oxygen consumption rate

Cells were cultured in 24-well Seahorse XFe24 plates (Agilent, Santa Clara, CA) and were incubated at 37 °C under a humidified 5% CO_2_ atmosphere. Differentiated mouse myotubes were washed twice per day for 5 days with XF base medium (Agilent) and incubated with XF base medium at 37 °C without CO_2_ for 12 h. To determine glycolysis and glycolytic rate, three initial measurements of extracellular acidification rate (ECAR) were made using the XFe24 Seahorse analyzer, and then 10 mM glucose, 4 µg/mL oligomycin, and 100 mM 2DG were sequentially injected and nine additional measurements were performed (three during each condition). To determine oxygen consumption rate (OCR), other cells were initially measured at three time points during basal respiration, and then 4 µg/mL oligomycin (ATP synthase inhibitor), 9 µM carbonyl cyanide 4-(trifluoromethoxy) phenylhydrazone (FCCP; uncoupler of oxidative phosphorylation), and 11 µM antimycin A/5 µM rotenone (blocks mitochondrial respiration) were sequentially injected. During each condition three measurements of OCR were made. Data were analyzed with Wave software (version 2.6.0).

### Enzyme activity

Activities of glycolytic enzymes hexokinase, phosphofructokinase, pyruvate kinase, and lactate dehydrogenase were measured from ~35 mg of tissue (5 mg for lactate dehydrogenase and ~10 mg for each other enzyme activity assay) collected from muscle biopsies of acutely resistance exercised and resistance exercise-trained participants using the hexokinase activity assay kit (cat. no. ab136957), 6-phosphofructokinase activity assay kit (cat. no. ab155898), and pyruvate kinase activity assay kit (cat. no. ab83432) from Abcam (Cambridge, MA), and lactate dehydrogenase (cat. no. MAK183) from Sigma-Aldrich (St. Louis, MO) using the manufacturer’s directions. Muscle biopsies from a subset of 14 of the 17 acutely exercised participants were used for hexokinase, phosphofructokinase, and lactate dehydrogenase activity assays due to sample availability and financial constraints. Additionally, from those 14 participants (5 male and 9 female), biopsies from 12 participants (5 male and 7 female) were used for pyruvate kinase activity assays for the same reasons. Among these two smaller cohorts used for acute exercise activity assay measures, the demographics (age, BMI, and recorded 1-RM) are not significantly different from our larger cohort of 17 participants (Supplemental Table 1). For the activity assays performed on resistance exercise-trained muscle biopsy samples, a cohort of 14 (7 younger and 7 older) of the 18 total participants from the original study were used due to sample limitations. Because of sample limitations, only 10 participants could be assayed for the lactate dehydrogenase activity assay from these participants (5 younger and 5 older). Hexokinase activity assay for C_2_C_12_ myotubes was analyzed using the manufacturer’s protocol (cat. no. ab136957).

### Image for GLUT4 translocation

Immunofluorescent detection of GLUT4 was conducted as previously described^69^. Myoblasts were washed with DPBS and then fixed with 4% formaldehyde for 10 min. Myoblasts were incubated in 0.1% Triton X-100 in PBS, washed with DPBS, and blocked with 1% BSA, all at room temperature (RT). Cells were incubated overnight with GLUT4 (cat. no. ab-654, Abcam) and dystrophin (cat. no. ab15277, Abcam) primary antibodies in 5% Goat serum and washed with DPBS. Appropriate fluorescent secondary antibodies (Alexa Fluor 488 goat anti-rabbit IgG (A11034) or Alexa Fluor 594 goat anti-mouse (A11005)) in PBS were incubated for 30 min at RT. Cells were then washed and incubated with DAPI for 5 min. Myoblasts were washed three times and mounted with a coverslip. Cells were imaged using a Nikon 100X oil immersion lens.

### Analysis using C_2_C_12_ myotube

We obtained microarray data that was collected using C_2_C_12_ myotubes overexpressing PGC-1α4 or PGC-1α1. This data was obtained from the publicly accessible online supplemental material from a previously published paper by Jorge L. Ruas (Karolinska Institutet, Stockholm, Sweden)^19^. We analyzed the change in glycolytic gene expression with these two mouse muscle cell models. In brief, raw gene expression files were obtained for Gene Expression Omnibus project#GSE42473. Data files were processed using Partek Genomics Suite (version 7.0) software configured to use default data processing parameters corresponding to the Affymetrix Mouse Genome 430 2.0 array. Differential expression analysis of muscle genes in PGC-1α4 or PGC-1α1 myotubes was performed by comparing each genotype separately with muscle gene expression of cells expressing a GFP empty vector, following Partek’s guidelines. Genes that had an adjusted *P*-value < = 0.05 and an absolute log2(fold-change) ≥ 0.5 (wherein 0.0 corresponds to no change) were considered as statistically significant.

### Plasma lactate

Concentration of plasma lactate was measured using 50 µl of plasma by gas chromatograph mass spectrometry (GC/MS) done against a 7-point calibration curve that underwent the same derivatization with internal standard, as previously described^70^.

### Statistical analysis

Statistical analyses of all data sets except gene differential expression were conducted using Prism 8 software (GraphPad, San Diego, CA). Briefly, results of all variables are presented as means ± SEM. The paired and unpaired Student’s *t*-test and one-way ANOVA was performed according to the study design. A Tukey’s test was used for post hoc analyses to determine significant differences between individual groups. Gene RPKM values were utilized when correlating, via Spearman’s method, PGC-1α4 expression with that of selected glycolysis-related genes in skeletal muscle from sedentary participants measured by RNA-Seq.

### Reporting summary

Further information on research design is available in the Nature Research Reporting Summary linked to this article.

## Supplementary information

Supplementary Information

Reporting Summary

## Data Availability

All data supporting the findings described in this manuscript are available in the article and in the Supplementary Information and from the corresponding author upon reasonable request. Source data are provided with this paper. RNA-Seq data have been previously deposited to GEO under accession number GSE97084. Source data are provided with this paper.

## Source data

Source data
