# Supplementary material for: Enhancement of anaerobic glycolysis – a role of PGC-1α4 in resistance exercise
Source: Nat Commun. 2022 Apr 28;13:2324. doi: 10.1038/s41467-022-30056-6 (PMC9050893; doi:10.1038/s41467-022-30056-6)
Supplement: Supplementary file 1 — Supplementary Information [file 41467_2022_30056_MOESM1_ESM.pdf]

Supplementary Information

	Males (n=8)		Females (n=9)	
	mean	SD	mean	SD
age	29.4	5.0	33.7	9.6
Height (cm)	176.9	7.7	165.5	6.8
Weight (kg)	84.6	14.3	72.5	12.3
BMI	27.0	3.6	26.4	3.9
1-RM	214.8	73.2	115.0	21.9
1-RM/kg	2.5	0.6	1.6	0.5

Supplemental Table 1: Acute exercise study participant characteristics.

qRT-PCR primers for gene expression

Gene	Forward (5' - 3')	Reverse (5' - 3')
18S	GTAACCCGTTGAACCCCATT	CCATCCAATCGGTAGTAGCG
PGC-1α	CAGCCTCTTTGCCCAGATCTT	TCACTGCACCACTTGAGTCCAC
PGC-1α1	ATGGAGTGACATCGAGTGTGCT	GAGTCCACCCAGAAAGCTGT
PGC-1α4	TCACACCAAACCCACAGAGA	CTGGAAGATATGGCACAT
PPARβ	CTCTATCGTCAACAAGGACG	GTCTTCTTGATCCGCTGCAT
HKII	GAAGATGCTGCCCACCTTTG	CACCCAAAGCACACGGAAGT
GFPI	CCCTATGACCAGTACCTGCAC	TTCCCATTGGACTCCATGTC
PFK1	GAGTGACTTGTTGAGTGACCTCCAGAAA	CACAATGTTCAGGTAGCTGGACTTCG
ALDOB	AGCCTCGCTATCCAGGAAAACG	TGGCAGTGTTCCAGGTCATGGT
PKM	TCACTCCACAGACCTCATGG	GAAGATGCCACGGTACAGGT
CD36	CAGGTCAACCTATTGGTCAAGCC	GCCTTCTCATCACCAATGGTCC
SLC25A20	ACCGAGTTTGCCTGGACAACCT	CCCAAAGAAGCACACGGCAAAC
NDUFA9	GCCCATTTGAGCCCTGGATAAC	GCCTTGAGTTCCAGTGGTGTTG
SDHB	GCAGTCCATAGAAGAGCGTGAG	TGTCTCCGTTCCACCAGTAGCT
CYCS	AAGGGAGGCAAGCACAAGACTG	CTCCATCAGTGTATCCTCTCCC
UQCRC2	CCGTGGAATTGAAGCAGTTGGTG	CTGTGGTGACATTGAGCAGGAAC
ATP5A1	GCTCCTTACTCTGGCTGTTCCA	GCGGAGCAACAGAGACATCTGA
COX4i1	TCGGTTTCACCGCGCTCGTTAT	TGTCCAGCATCCTCTTGGTCTG

Supplemental Table 2: qPCR primers used for gene expression in human muscle biopsies are shown.

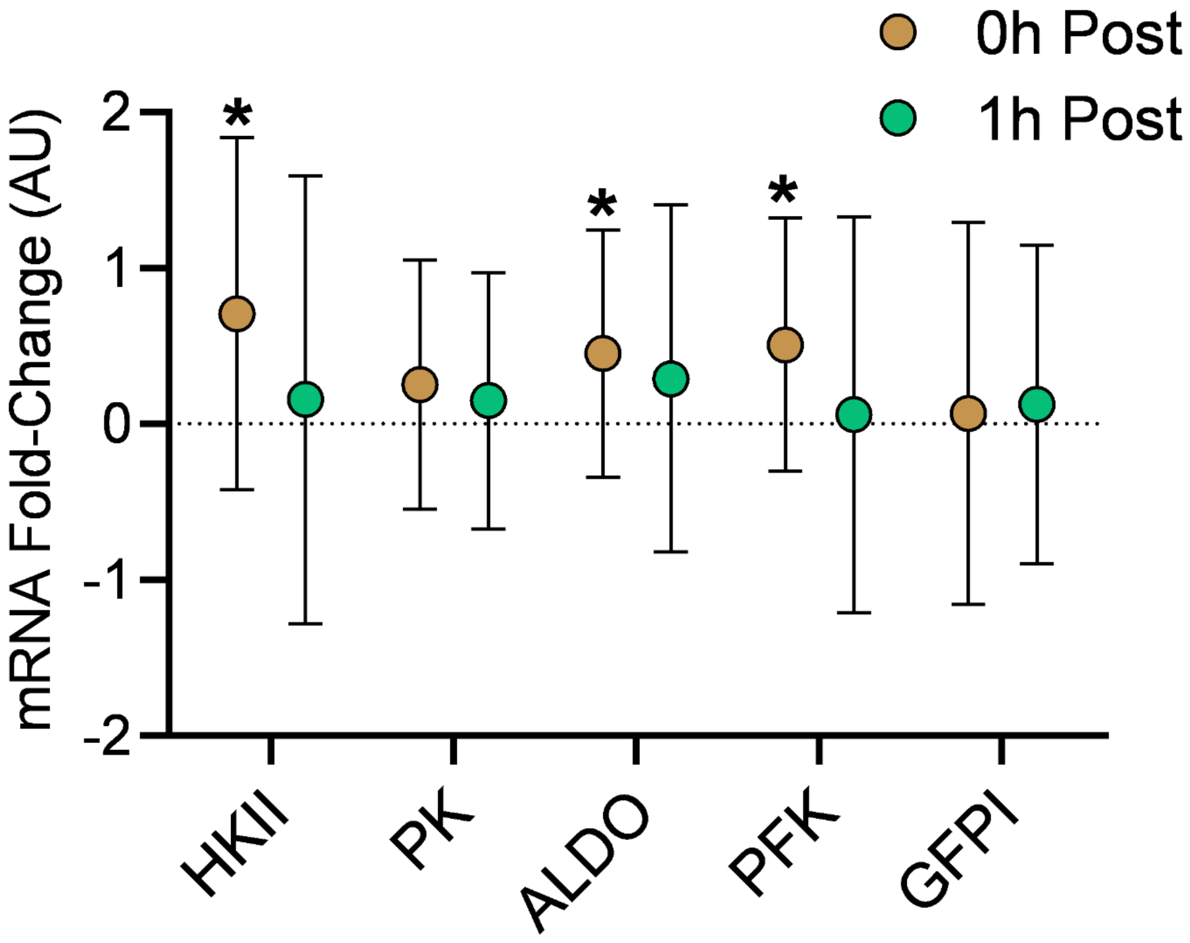

Supplemental Figure 1. Glycolytic enzyme skeletal muscle mRNAs (n=16) were analyzed using qPCR after a single session of resistance exercise and data are presented as the difference between the exercised sample and its time-matched sedentary control for each post-exercise time-point (0h and 1h). HKII (Hexokinase 2), PK (Pyruvate Kinase), ALDO (Aldolase), PFK (Phosphofructokinase), GFPI (Glucose-6-Phosphate isomerase). Paired two tailed t-tests were used to compare post=-exercise values versus sedentary control values from the same leg. *$p < 0.05$ versus sedentary leg. Values are expressed as mean ± SD. Significant labeled p- left to right are as follows: 0.024, 0.038, and 0.025

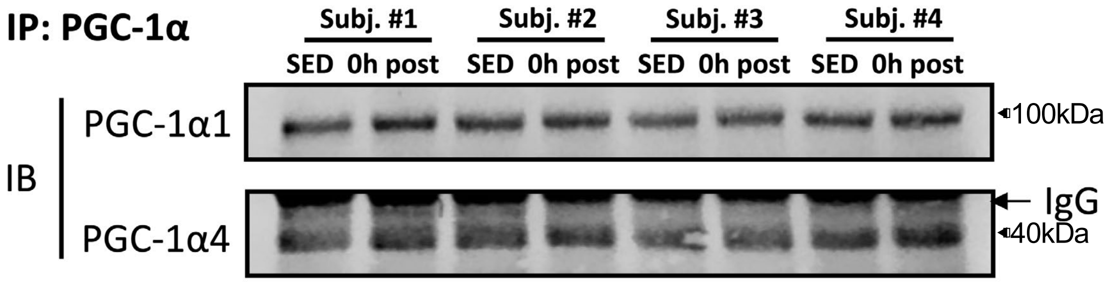

Supplemental Figure 2. Muscle biopsies were obtained before (SED) and immediately after (0h Post) acute one-legged resistance exercise in two participants. In two other participants muscle biopsies were obtained from the sedentary leg and from the exercised leg approximately 1h following the resistance exercise bout. Total-PGC-1α was immunoprecipitated and then PGC-1α1 and PGC-1α4 were immunoblotted. Experiment was repeated using the same sample lysates with similar results.

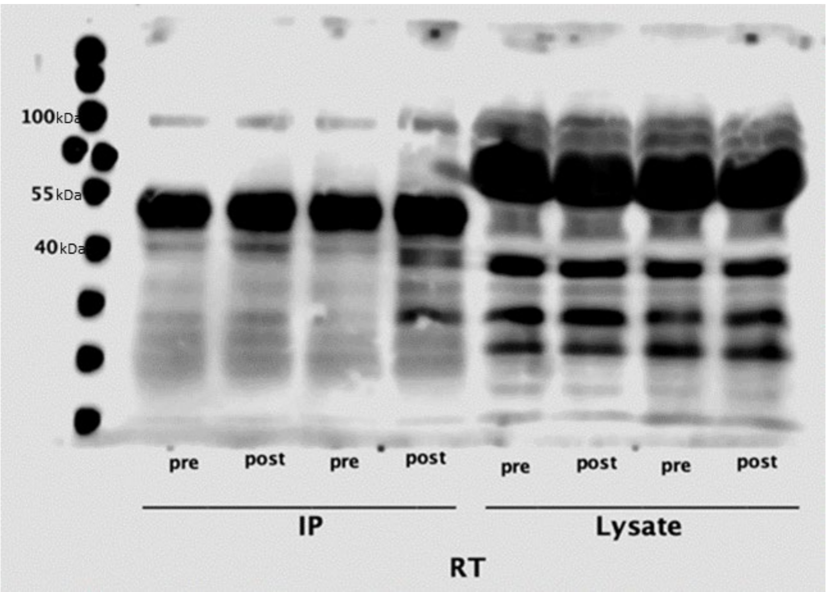

Supplemental Figure 3: Full blot image from Figure 2H is provided with the input controls (Lysate).

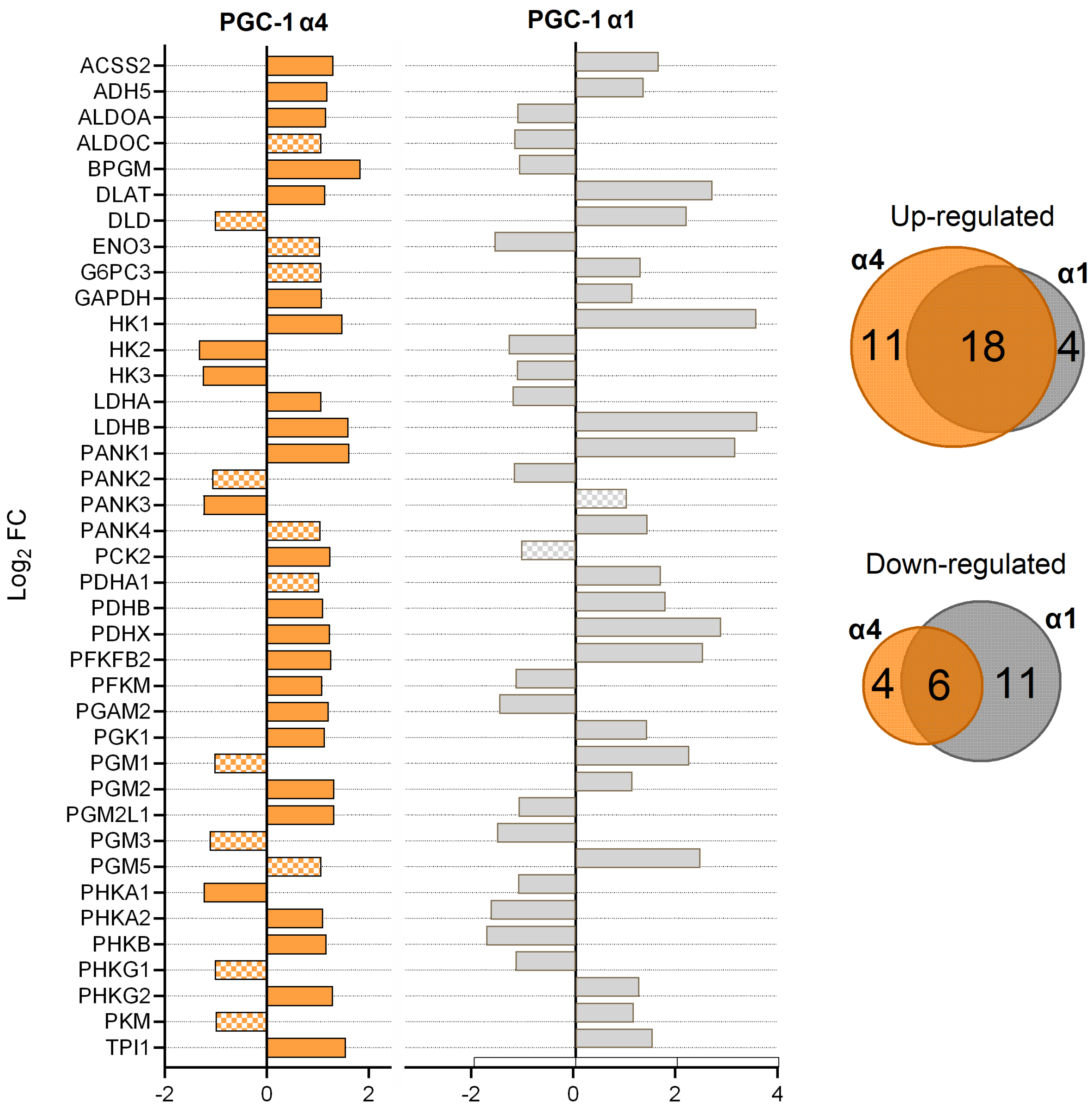

Supplemental Figure 4: C_2C_{12} myotubes overexpressing PGC-1α4 (orange) or PGC-1α1 (grey) were analyzed by microarray for glycolysis-related genes in muscle from previously reported data [19] . Glycolysis pathway-related genes were collected according to the list of Kyoto Encyclopedia of Gene and Genomes. Change in glycolysis pathway gene expression in the muscle cells overexpressing PGC-1α4 and PGC-1α1 are shown in the bar graphs. Shaded bars indicate significantly different from control values ($P < 0.05$). Ven-diagrams displayed to the right highlight the number of up-regulated or down-regulated genes with each genotype. Experiments were performed in triplicate as previously indicated [19]. Values are expressed as Log_2 FC.

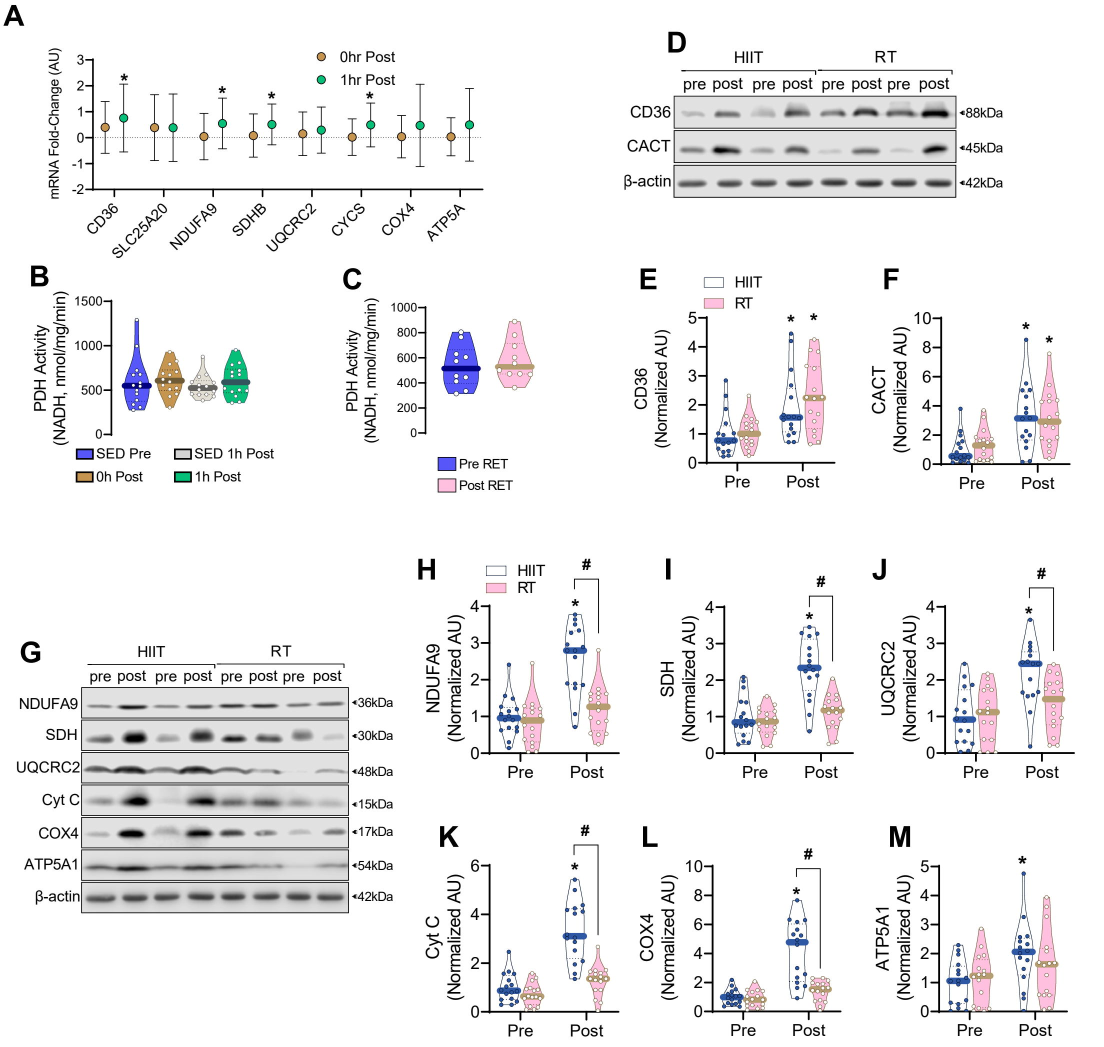

Supplemental Figure 5. (**A**) mRNA expression was determined in muscle by qPCR from acutely resistance exercised participants. Data are represented as the difference in mRNA expression from the exercised muscle and the mRNA expression from its time-matched sedentary control muscle in the contralateral leg (n=16). Paired two tailed t-tests were used. (**B-C**) Pyruvate dehydrogenase activity was measured in muscle samples from acutely resistance exercised participants (n=14) and resistance exercise trained participants (n=10). One-Way ANOVA was used with multiple comparisons for panel B, and paired two tailed t-test was used for panel C. (**D-M**) The abundance of proteins related to fat metabolism (**D-F**) and mitochondrial enzymes (**G-M**) were determined in skeletal muscle before (pre) and after (post) 12 weeks of resistance exercise training (RET) or aerobic exercise training (HIIT; high intensity interval training). Representative blots for each protein (**D, G**) and quantification of the relative change (**E, F, H-M**) after training for fatty acid translocase (CD36), Carnitine-acylcarnitine translocase (CACT), NADH Dehydrogenase 1 alpha subcomplex subunit 9(NDUFA9; complex I), Succinate Dehydrogenase (SDH; complex II), ubiquinol-cytochrome c reductase core protein 2 (UQCRC2; complex III), Cytochrome c (Cyt c), Cytochrome c oxidase subunit 4 (COX4; complex IV), and ATP synthase α (ATP5A1) are displayed (n = 16 per group). One-Way

ANOVAs were used with multiple comparisons. *$p < 0.05$ versus pre-exercise, #$p<0.05$ post HIIT versus post RT. Values are expressed as individual data points, Log_2 FC, or mean ± SD. Significant labeled p-values in each panel from left to right are as follows: panel A = 0.036, 0.041, 0.020, and 0.035; panel E = 0.001 and <0.001; panel F = <0.001 and <0.001; panel H = <0.001 and <0.001; panel I = <0.001 and <0.001; panel J = <0.001 and 0.006; panel K = <0.001 and <0.001; panel L = <0.001 and <0.001; panel M = <0.001.

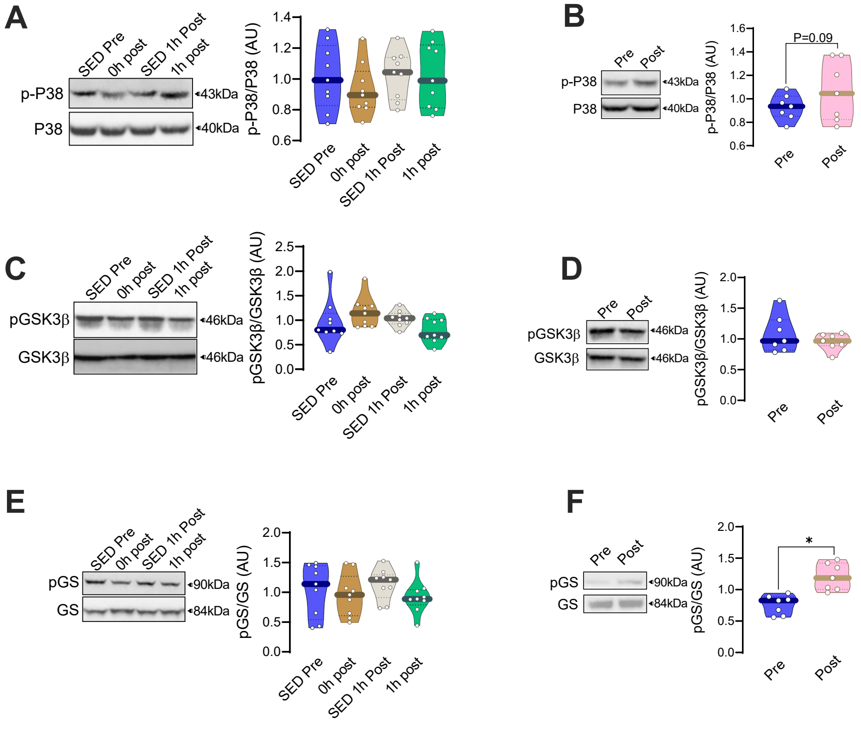

Supplemental Figure 6: (**A, C, & E**) Acute resistance exercise effect on P38MAPK, GSK3β, and glycogen synthase (GS) phosphorylation in skeletal muscle. One-Way ANOVAs were used with multiple comparisons. (**B, D, & F**) Resistance exercise training effect on P38MAPK, GSK3β, and glycogen synthase (GS) phosphorylation in skeletal muscle. Paired two tailed t-tests were used. * $p<0.05$. Values are expressed as individual data points. Significant labeled p-values in each panel are as follows: panel B = 0.09; panel F = <0.001.

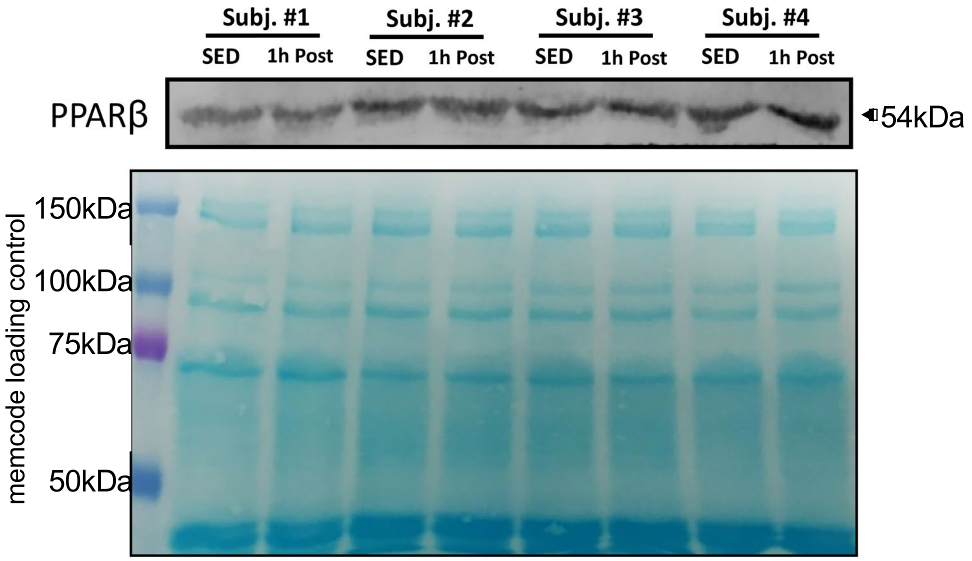

Supplemental Figure 7: PPARβ was immunoblotted from muscle biopsies obtained before (pre) and 1h after (1h Post) an acute bout of resistance exercise in 4 participants. Memcode protein staining was used to show equal loading. Experiment was performed once.

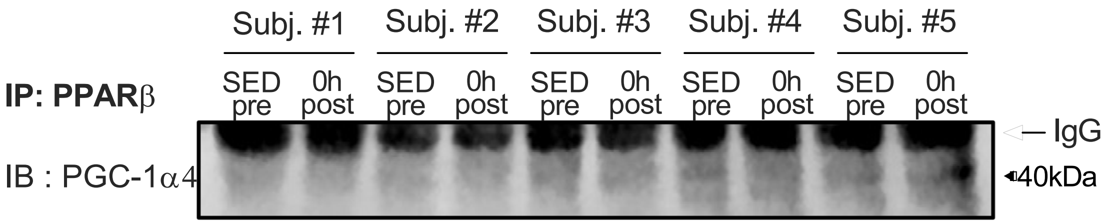

Supplemental Figure 8: PPARβ was immunoprecipitated from muscle biopsies obtained before (SED Pre) and immediately after (0h Post) an acute bout of resistance exercise in 5 participants. Following immunoprecipitation, samples were immunoblotted for PGC-1α4 and weak signal was found in all samples.

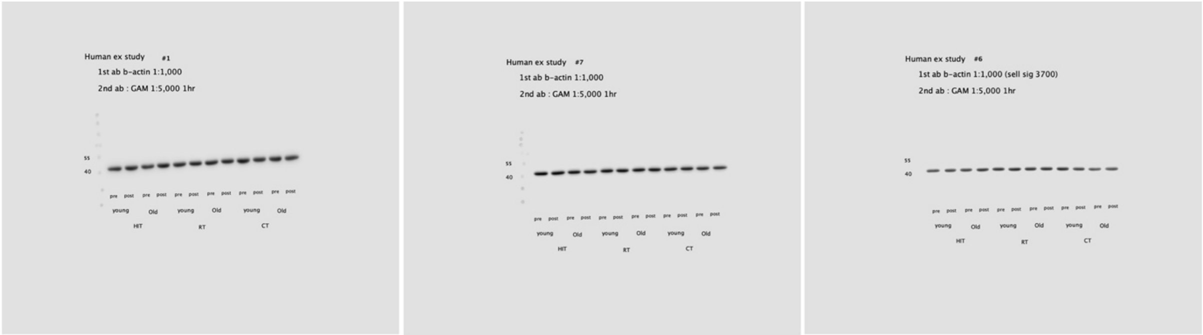

Supplemental Figure 9: Full blot images of β-Actin from PGC-1α blots (Figure 2G). Images show that β-Actin was unaltered by three different training modes (HIIT, high-intensity interval training; RT, resis
