# Supplementary material for: Enhancement of anaerobic glycolysis – a role of PGC-1α4 in resistance exercise
Source: Nat Commun. 2022 Apr 28;13:2324. doi: 10.1038/s41467-022-30056-6 (PMC9050893; doi:10.1038/s41467-022-30056-6)
Supplement: Supplementary file 2 — Reporting Summary [file 41467_2022_30056_MOESM2_ESM.pdf]

nature portfolio

Corresponding author(s): DBPR NCOMMS-21-17151C

Last updated by author(s): Apr 13, 2022

Reporting Summary

Nature Portfolio wishes to improve the reproducibility of the work that we publish. This form provides structure for consistency and transparency in reporting. For further information on Nature Portfolio policies, see our Editorial Policies and the Editorial Policy Checklist.

Statistics

For all statistical analyses, confirm that the following items are present in the figure legend, table legend, main text, or Methods section.

n/a	Confirmed	
☐	☒	The exact sample size (*n*) for each experimental group/condition, given as a discrete number and unit of measurement
☐	☒	A statement on whether measurements were taken from distinct samples or whether the same sample was measured repeatedly
☐	☒	The statistical test(s) used AND whether they are one- or two-sided<br>*Only common tests should be described solely by name; describe more complex techniques in the Methods section.*
☐	☒	A description of all covariates tested
☐	☒	A description of any assumptions or corrections, such as tests of normality and adjustment for multiple comparisons
☐	☒	A full description of the statistical parameters including central tendency (e.g. means) or other basic estimates (e.g. regression coefficient) AND variation (e.g. standard deviation) or associated estimates of uncertainty (e.g. confidence intervals)
☐	☒	For null hypothesis testing, the test statistic (e.g. *F*, *t*, *r*) with confidence intervals, effect sizes, degrees of freedom and *P* value noted<br>*Give P values as exact values whenever suitable.*
☒	☐	For Bayesian analysis, information on the choice of priors and Markov chain Monte Carlo settings
☒	☐	For hierarchical and complex designs, identification of the appropriate level for tests and full reporting of outcomes
☐	☒	Estimates of effect sizes (e.g. Cohen's *d*, Pearson's *r*), indicating how they were calculated

Our web collection on statistics for biologists contains articles on many of the points above.

Software and code

Policy information about availability of computer code

Data collection	A full description of all data collection methods are described in the methods section
Data analysis	Prism 8 software (GraphPad, San Diego, CA); Chenomx NMR suite software (Chenomx, Edmonton, Canada); TruSeq SBS sequencing kit version 3 and HCS version 2.0.12.0 software; edgeR software (version 3.225)

For manuscripts utilizing custom algorithms or software that are central to the research but not yet described in published literature, software must be made available to editors and reviewers. We strongly encourage code deposition in a community repository (e.g. GitHub). See the Nature Portfolio guidelines for submitting code & software for further information.

Data

Policy information about availability of data

All manuscripts must include a data availability statement. This statement should provide the following information, where applicable:

- Accession codes, unique identifiers, or web links for publicly available datasets
- A description of any restrictions on data availability
- For clinical datasets or third party data, please ensure that the statement adheres to our policy

All data will be made fully accessible upon reasonable request

Field-specific reporting

Please select the one below that is the best fit for your research. If you are not sure, read the appropriate sections before making your selection.

- [x] Life sciences
- [ ] Behavioural & social sciences
- [ ] Ecological, evolutionary & environmental sciences

For a reference copy of the document with all sections, see nature.com/documents/nr-reporting-summary-flat.pdf

Life sciences study design

All studies must disclose on these points even when the disclosure is negative.

Sample size	Sample sizes were determined based off previously performed power calculations and sample availability
Data exclusions	No data were excluded
Replication	When reasonable, data were run in duplicate and confirmed for reproducibility. Other reproducibility was determined for some data based on previous findings and previous use of the described methods. All attempts at replication were successful. This information has been included in the figure legends.
Randomization	Randomization is described in the methods when applicable. Allocation into experimental group was random.
Blinding	It is impossible to blind researchers to a participant performing acute exercise.

Reporting for specific materials, systems and methods

We require information from authors about some types of materials, experimental systems and methods used in many studies. Here, indicate whether each material, system or method listed is relevant to your study. If you are not sure if a list item applies to your research, read the appropriate section before selecting a response.

Materials & experimental systems

n/a	Involved in the study	
[ ]	[x]	Antibodies
[ ]	[x]	Eukaryotic cell lines
[x]	[ ]	Palaeontology and archaeology
[x]	[ ]	Animals and other organisms
[ ]	[x]	Human research participants
[x]	[ ]	Clinical data
[x]	[ ]	Dual use research of concern

Methods

n/a	Involved in the study	
[x]	[ ]	ChIP-seq
[x]	[ ]	Flow cytometry
[x]	[ ]	MRI-based neuroimaging

Antibodies

Antibodies used	Antibodies used for specific detection of MEF2A (cat. no. 9736), AMPKα (cat. no. 2793), α-tubulin (cat. no. 2125), phospho-AMPKα Thr-172(cat. no. 2531), phospho-AS160 (cat. no. 4288), AS160 (cat. no. 2447), phospho-Glycogen Synthase (cat. no. 3891), Glycogen Synthase (cat. no. 3893), phospho-GSK3β (cat. no. 5558), GSK3β (cat. no. 12456), p38 MAPK (cat. no. 8690), and phospho- p38 MAPK (cat. no. 4511) were from Cell Signaling Technologies (Danvers, MA); PGC-1α (cat. no. 516557) from Millipore (Billerica, MA); PPARβ (cat. no. PA5-29678), NDUFA9 (cat. no. 459100), COX4 (cat. no. A21348), UQCRC1 (cat. no. 459140), and ATP5A1 (cat. no. 459240) were from Thermo Fisher Scientific (Houston, TX); PFK (cat. no. SC-166722), PDK4 (cat. no. SC-130841), SOD2 (cat.no. sc-137254), Catalase (cat. no. sc-271803), and NRF-1 (cat. no. SC-23624) were from Santa Cruz Biotechnology (Santa Cruz, CA); β-actin (cat. no. A5441) was from Sigma Aldrich; GLUT4 (cat. no. ab-654), Na+/K+ ATPase (cat. no. ab76020), LCAD (cat. no. 196655), and UQCRC2 (cat. no. ab14745) were from Abcam (Cambridge, MA); CD36 (cat. No. 18836-1-AP), LDHa (cat. no. 19987-1-AP), LDHb (cat. no. 14824-1-AP), Pyruvate Carboxylase (PC; cat. no. 16588-1-AP), and CACT (cat. no. 19363-1-AP) were from proteintech (Rosemont, IL); Succinate dehydrogenase (SDH; cat. no. 439300) was from Innovative Research; Cyt C (cat. no. 556433) was from BD Biosciences; PHKA1 (cat. no. GTX109401) was from GeneTex (Alton, CA); CPT1M (cat. no. CPT1M11-A) from Alpha diagnostic (San Antonio, TX); and secondary antibodies such as donkey anti-mouse (cat. no. 715-035-150), donkey anti-rabbit (cat. no. 711-035-152), and streptavidin (cat. no. 016-030-084) were obtained from Jackson Immunoresearch Laboratories (West Grove, PA). The dilution for all primary antibodies was 1:1,000. The dilution for all secondary antibodies was 1:10,000. This information is not in the manuscript.
Validation	There are dozens of antibodies that we have used. The manufacturer has provided validation information. Some have been validated using KO cell lines, some have not been as rigorously validated, but show up at the predicted molecular weight without ample non-specific binding.

Eukaryotic cell lines

Policy information about cell lines

Cell line source(s)	C2C12 mouse myotubes ATCC
Authentication	The C2C12 cell line used is commercially available through ATCC and all information about the C2C12 cell line that is sold by ATCC can be found on their website.
Mycoplasma contamination	The cells did not test positive for myoplasma contamination
Commonly misidentified lines (See ICLAC register)	none

Human research participants

Policy information about studies involving human research participants

Population characteristics	All participants have pre-exercise vs post-exercise values that were obtained, serving as their own internal control. As stated in the manuscript, participants in the acute exercise study are between the ages of 18-55 years old, with a BMI between 21-32 kg/m2. Approximately half the participants are male and half female. All specific information about participants can be found in the manuscript.
Recruitment	Participants were recruited by physical and online flyers in Rochester, Minnesota. There are no known self-selection biases.
Ethics oversight	All studies were approved by the Mayo Clinic Institutional Review Board and all participants gave their informed consent to participate in the studies. The study design and conduct complied with all relevant regulations regarding the use of human study participants and was conducted in accordance with the criteria set by the Declaration of Helsinki.

Note that full information on the approval of the study protocol must also be provided in the manuscript.
